# Identifying the Mental Health Research Priorities in Rural Settings, With Implications for Coastal Communities: A Rapid Evidence Synthesis

**DOI:** 10.1111/ajr.70171

**Published:** 2026-03-20

**Authors:** Viet‐Hai Phung, David Nelson, Ros Kane, Kyla Pennington, Joseph Akanuwe, Harriet Moore, Robert Dean, Russell Roberts, Derek Ward, Jaspreet Phull, Tracy McCranor, Colin Hopkirk, Jon Mansfield, Richard Morriss, Mark Gussy, Dave Dawson, Nima Moghaddam

**Affiliations:** ^1^ Lincoln Institute for Rural and Coastal Health University of Lincoln Lincoln UK; ^2^ School of Health and Care Sciences University of Lincoln Lincoln UK; ^3^ Department of Psychology University of York York UK; ^4^ Community and Health Research Unit University of Lincoln Lincoln UK; ^5^ Lincoln School of Creative Arts, College of Arts Social Sciences and Humanities University of Lincoln Lincoln UK; ^6^ Manna Institute, Equally Well, School of Business Charles Sturt University Bathurst New South Wales Australia; ^7^ Lincolnshire County Council Lincoln UK; ^8^ Lincolnshire Partnership NHS Foundation Trust Lincoln UK; ^9^ Every One Lincoln UK; ^10^ Faculty of Medicine and Health Sciences, Institute of Mental Health University of Nottingham Nottingham UK; ^11^ School of Psychology, Sport Science and Wellbeing University of Lincoln Lincoln UK

**Keywords:** coastal health, mental health, priority setting, rapid review, rural health

## Abstract

**Introduction:**

There appears to be a spatial mismatch in rural communities between demand for and uptake of mental health services. There is currently no existing evidence synthesis of mental health research priorities pertaining explicitly to rural and coastal contexts.

**Objective:**

The rapid review aimed to identify and map existing international evidence on rural and coastal mental health research priorities.

**Design:**

A rapid systematic review was conducted, consistent with guidelines from the Cochrane Rapid Review Methods Group and PRISMA. Keywords and subject headings were searched in PubMed and PsycINFO. Supplementary searching was performed in Google Scholar. Data were extracted using an adapted version of the REPRISE framework. Content analysis was conducted to establish priorities.

**Findings:**

1285 studies were screened and 20 publications included (Australia *n* = 8, USA *n* = 9, UK *n* = 2, no geographic focus *n* = 1). The content analysis grouped the priorities into seven categories: (1) interventions; (2) space and place; (3) stakeholder engagement; (4) improving understanding; (5) standardising data and terminology; (6) outreach; and (7) collaboration. Within these categories, there were 16 priorities and 53 sub‐priorities. No evidence focused on mental health research priorities in coastal contexts.

**Discussion:**

Future rural mental health research requires stronger collaboration between relevant stakeholders to reflect local needs. Participatory research is key to achieving that. There was no mental health research priority setting exercise that accounted for the coastal context, highlighting a notable gap.

**Conclusion:**

The findings can inform how rural and coastal mental health research proceeds at a local, national, and international level.

## Abstract (highlights)

**What Does This Paper Add?:**

- This rapid evidence review is the first to synthesise the international evidence on mental health research priorities in rural settings.
- The findings can support rural mental health researchers to contextualise their own research priorities within the wider extant literature.
- There is an urgent need for a mental health research priority setting exercise unique to the coastal context.

**What Is Already Known on This Subject?:**

- There is growing concern around the availability of both health and mental health services for rural, remote, and coastal communities.
- Mental health research priority setting exercises can support the allocation of resources towards the most pressing and impactful research questions.

## 1 Introduction

There is increasing evidence that the physical and natural environment in which people live and work can impact on their mental health and wellbeing. There is growing concern around the availability of both health and mental health services for rural communities. Rural residents receive mental health treatment less frequently and from professionals with less specialised training compared to their urban counterparts [1, 2, 3, 4].

Alongside rural communities, many of the UK's coastal areas face considerable health and social challenges. Their populations suffer from relatively poor mental and physical health, much of which has been driven by decades of socio‐economic decline [5]. Historically, policy has given limited focus to the health challenges faced by coastal communities [6]. However, the bespoke health challenges faced by coastal communities have been acknowledged by the Chief Medical Officer for England in his annual reports in 2021 [7] and 2023 [8]. Despite recent policy recognition, coastal mental health research remains an under‐explored area internationally [9, 10, 11].

Research priority setting exercises are useful as they can support researchers and policymakers with the allocation of resources towards the most pressing and impactful research questions [12]. The GLiMHR (Guiding Lincolnshire in Mental Health Research) study aims to identify priorities for mental health research in the predominantly rural and coastal county of Lincolnshire in the East Midlands region of England. Lincolnshire faces significant mental health challenges, especially in its rural and coastal areas. Lincolnshire has one of the highest suicide rates in the East Midlands [13] and includes rural and coastal towns. It has disproportionately high levels of people with severe mental health needs.

Factors, such as socio‐economic deprivation, high depression prevalence, and heavy use of antidepressants, are particularly problematic in rural and coastal communities in Lincolnshire [14, 15]. There is, therefore, a mismatch between the region's high need for and the availability of mental health support and solutions.

To support the GLiMHR study, we undertook a rapid evidence synthesis to contextualise and align any local priority setting exercise with the wider international research evidence. This rapid evidence synthesis aimed to:

- Identify and map the national and international evidence on mental health research priorities in rural and coastal settings.

## 2 Methods

### 2.1 Study Design

We adopted a rapid review approach. Rapid reviews are evidence syntheses where components of a systematic review are omitted or simplified to produce findings in a timely manner [16]. This is now considered a key component of evidence synthesis, alongside systematic reviews, scoping reviews, and realist reviews [17], although there is still no consensus about how a rapid review should be conducted or defined [16]. However, there is agreement that they are a streamlined approach to evidence synthesis where there is a need to generate evidence quickly.

The primary aim of this review was to generate evidence quickly on rural mental health research priorities globally that could then inform a local priority setting exercise in a rural and coastal UK setting. With a view to the timeliness and need for outcomes, it was decided that a quality appraisal of the included studies was not deemed appropriate and the omission of specific review components, such as a quality assessment, is in line with other rapid reviews.

While not having a quality appraisal allowed the rapid evidence review to capture a broader range of studies, and with it, a broader range of data, there is a risk that lower quality publications are included. This rapid review was conducted in line with guidance from the Cochrane Rapid Reviews Methods Group [18] and reported using the PRISMA checklist (see Supporting Information S2) [19]. The protocol was registered on the Open Science Framework [https://osf.io/hsjfb/] and was conducted from September–November 2024.

### 2.2 Eligibility Criteria

Articles were eligible for inclusion if they reported findings on priorities, recommendations, or agenda setting with regard to mental health research in rural, remote, and coastal areas (Table 1). Before we proceed, it is important to acknowledge the lack of agreement over what these three terms mean. The definitions of each of these terms appear to vary by geographical context in which they are used. This could include research studies, as well as commentary and expert opinion pieces. Articles needed to have an explicit focus on mental health, although articles which reported broader rural health research priorities that included mental health were included. Articles were restricted to those published in English language, peer‐reviewed academic journals. Grey literature and unindexed regional studies were excluded. There were no restrictions on the date of publication.

**TABLE 1:** Article eligibility criteria.

Domain	Inclusion criteria
*Population*	People involved with mental health care (includes patients, caregivers, professionals clinical, non‐clinical, community based) and researchers.
*Concept*	Articles that reported some data on mental health priorities/agenda setting/recommendations in rural, and coastal settings OR articles that reported rural health (including coastal) priorities/agenda setting/recommendations that explicitly relate to mental health.
*Context*	Mental health care/research (clinical and non‐clinical) in rural, and coastal settings.
*Type*	In English language, peer‐reviewed publications. Article type included: commentary, expert opinion pieces, research studies and explicit priority/agenda setting exercises.
*Publication date*	No restrictions.

### 2.3 Search Strategy

A combination of keywords and medical subject headings was searched in PubMed and PsycINFO on 18th September 2024 to locate relevant literature. These databases were chosen for their strong coverage of clinical and mental health services research, with indexing of key journals in mental health, public health, and global health. We also conducted supplementary searching using keywords in Google Scholar. The primary search strategy and syntax were developed and refined by VHP and DN. The searches were broken down conceptually by (1) mental health related terms, (2) rural‐ and coastal‐related terms and (3) research priority related terms. The syntax used in PubMed can be found in Table 2.

**TABLE 2:** PubMed syntax.

Concept	Syntax
*Mental health*	((((((((‘Mental Health’[Mesh]) OR (‘Mental Health’)) OR (‘Mental health servic*’)) OR (‘Mental health need*’)) OR (‘Mental health issue*’)) OR (‘Mental health provision’)) OR (‘Mental health access’)) OR (‘Mental health priorit*’)) OR (‘Mental health research’)
*Rurality*	(((((((‘Rural Health’[Mesh]) OR (‘Rural Health’)) OR (Rural*)) OR (Coast*)) OR (‘Rural mental health’)) OR (‘Rural health services delivery’)) OR (‘Rural health services’ [Mesh])) OR (‘Rural health services’)
*Research priorities*	((((‘Research’[Mesh]) OR (‘research priorit*’)) OR (‘research agenda’)) OR (‘Priority setting’)) OR (‘Agenda setting’)

### 2.4 Study Selection

**FIGURE 1:**
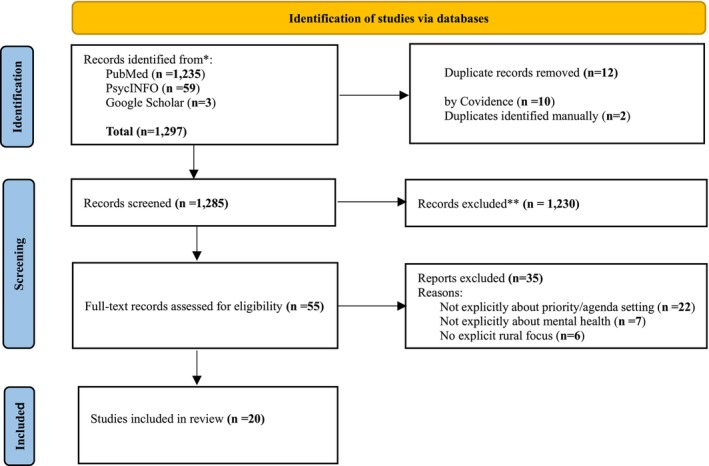
PRISMA flow diagram. *Source*: Moher et al. [19].

Data files from the searches were imported into Covidence software to facilitate management and screening [20]. Covidence automatically removes duplicate articles, although this was also manually checked where some duplicates were missed. Titles and abstracts were screened independently by VHP and DN. Where it was unclear from the title and abstract review whether the article met the eligibility criteria, the article automatically progressed to full‐text screening (Figure 1).

Full‐text articles were also screened independently by two reviewers, VHP and DN. Conflicts were resolved via discussion. At full‐text screening, reasons for exclusion were documented (e.g., no explicit rural or coastal focus, did not explicitly report research priorities/agenda setting). Independent screening by title and abstract, as well as full‐text, are essential to minimise bias.

### 2.5 Data Extraction

The relevant data from the included articles were extracted and tabulated by VHP with quality checking and validation performed by DN. Information was extracted using a modified version of the Reporting guideline for health research priority setting with stakeholders (REPRISE) framework (see Supporting Information S3) [21]. This included the following: (1) author and year of publication; (2) title; (3) journal name; (4) geographic setting; (5) article type; (6) approach/method used to identify/develop rural mental health research priorities/recommendations; (7) summary of rural mental health research priorities/recommendations/agenda; and (8) other notes of relevance.

### 2.6 Data Synthesis and Analysis

Data were analysed and summarised descriptively, with article characteristics tabulated. This was accompanied by a narrative summary. A rapid content analysis was performed by VHP and DN on the identified priorities so we could report on the similarities and differences in rural mental health research priorities across the international literature. Categories, priorities, and sub‐priorities were then generated according to the frequency with which they occurred in the included publications.

### 2.7 Ethics

This was a desk‐based review of the readily available academic evidence, which involved no primary data collection. Therefore, ethical approval was not required.

## 3 Results

### 3.1 Search Results

The search produced 1297 records. After duplicates were removed, 1285 records were screened. We excluded 1230 records after title and abstract screening. Following this, 55 full‐text records were screened for eligibility, of which 35 were excluded. The primary reasons for exclusion included: not explicit regarding mental health research priority setting or agenda setting (*n* = 22), not explicitly pertaining to mental health (*n* = 7), not explicitly relating to rural or coastal settings (*n* = 6). A total of 20 articles met the eligibility criteria and were included in the review (Figure 1 and Table 3). Six of the 20 included publications described how priority setting was operationalised and which stakeholder groups were involved [22, 23, 24, 25, 26, 27]. These are incorporated into the supplementary adapted REPRISE framework table (see Supporting Information S3) [21].

**TABLE 3:** Included publications.

Author	Year	Title	Location	Publication type	Methodology	Groups involved	Rural	Coastal	Journal
Abraham et al. [28]	1994	Mental health of rural elderly: A research agenda for nursing	USA	Review	N/A	N/A	Yes	No	Issues in Mental Health Nursing
Baker et al. [22]	2004	Planning research in rural areas	Australia	Research	The workshop was interactive. Preliminary brainstorming to identify and prioritise topics. Followed by facilitated small group discussions and presentations to the whole group.	Health professionals from aged care and a private hospital, a hospice, and Indigenous Health Service. Police. City Council. Self‐employed GPs. Psychologists. Consumer representatives.	Yes	No	Rural and Remote Health
Boyd and Parr [29]	2008	Social geography and rural mental health research	UK	Review	N/A	N/A	Yes	No	Rural and Remote Health
Carpenter‐Song and Snell‐Rood [30]	2017	The changing context of rural America: A call to examine the impact of social change on mental health and mental health care	USA	Review	N/A	N/A	Yes	No	Psychiatric Services
Eley and Barker [23]	2007	Rural and remote health research: Key issues for health providers in Southern Queensland	Australia	Research	The workshops used the nominal group technique to identify what participants thought were key health issues in their locations.	Participants from organisations directly involved with health care were complemented by representatives from local government, the police service and church groups.	Yes	No	The Australian Journal of Rural Health
Fraser et al. [31]	2002	Does one size really fit all? Why the mental health of rural Australians requires further research	Australia	Review	N/A	N/A	Yes	No	The Australian Journal of Rural Health
Handley et al. [32]	2011	Urban–rural influences on suicidality: Gaps in the existing literature and recommendations for future research	USA	Review	N/A	N/A	Yes	No	The Australian Journal of Rural Health
Hartley et al. [33]	2002	Behavioural health: Setting the rural health research agenda	USA	Review	N/A	N/A	Yes	No	The Journal of Rural Health
Hauenstein [34]	2014	Building the rural mental health system: from de facto system to quality care	USA	Book chapter	N/A	N/A	Yes	No	Annual Review of Nursing Research
Hourihan and Kelly [35]	2006	National health policy: What does this mean for rural mental health research?	Australia	Editorial	N/A	N/A	Yes	No	The Australian Journal of Rural Health
Judd [36]	2006	Progressing the agenda for rural mental health research	Australia	Editorial	N/A	N/A	Yes	No	Rural and Remote Health
Judd et al. [37]	2002	The mental health of rural Australians: Developing a framework for strategic research	Australia	Review	N/A	N/A	Yes	No	The Australian Journal of Rural Health
Keller et al. [38]	1999	A rural mental health research agenda: Defining context and setting priorities	USA	Review	N/A	N/A	Yes	No.	The Journal of Rural Health
McAllister et al. [24]	2012	Determining mental health research priorities in a Queensland region: An inclusive and iterative approach with mental health service clinicians, consumers and carers	Australia	Research	Data were collected using a modified Delphi technique, three rounds of surveys, field notes and research team reflection.	Overall, the research focus groups included 47 clinicians, 49 consumers and 21 carers, totalling 117 participants.	Indirectly, yes	No	Advances in Mental Health
Perkins et al. [25]	2019	The Orange Declaration on rural and remote mental health	Australia	Research	Over 2 days, mental health researchers, academics, service providers, managers and commissioners identified problems and solutions: making use of national and international experience and evidence about service models, evidence from small‐scale local pilots and novel data analyses.	Over 2 days, mental health researchers, academics, service providers, managers and commissioners identified problems and solutions: making use of national and international experience and evidence about service models, evidence from small‐scale local pilots and novel data analyses.	Yes	No	The Australian Journal of Rural Health
Roberts et al. [26]	2024	International declaration on rural mental health research: 10 guiding principles and standards	International	Research	This paper describes how an international panel of rural mental health leaders adapted digital conferencing and the rapid synthesis and translation process (RSTP) to build consensus on core principles of rural mental health research and their application to policy and practice.	This paper describes how an international panel of rural mental health leaders adapted digital conferencing and the rapid synthesis and translation process (RSTP) to build consensus on core principles of rural mental health research and their application to policy and practice.	Yes	No	The Australian Journal of Rural Health
Rost et al. [39]	2002	Use, quality, and outcomes of care for mental health: The rural perspective	USA	Review	N/A	N/A	Yes	No	Medical Care Research and Review
Teague et al. [27]	2025	Informing mental health research priorities and design with rural and agricultural communities: a public involvement consultation case study	UK	Research	A multi‐methods approach was used for data collection for this case study: an online survey (*n* = 29) and qualitative community group discussions (*n* = 10). Findings are presented descriptively and analysed with content analysis to generate indicative research priorities and recommendations for future mental health research study design.	The core aim of the PPI consultation case study was to engage rural and agricultural community members of a rural mental health Voluntary, Community, and Social Enterprise (VCSE) organisation to share their views about local priorities for mental health research affecting their community, and to discuss perceptions on study design and promotional aspects of research studies which could influence their choice to participate.	Yes	No	Mental Health and Social Inclusion
Thorndyke [40]	2005	Rural women's health: A research agenda for the future	USA	Commentary	N/A	N/A	Yes	No	Women's Health Issues
Wagenfeld [41]	1990	Mental health and rural America: A decade review	USA	Review	N/A	N/A	Yes	No	The Journal of Rural Health

### 3.2 Article Characteristics

**FIGURE 2:**
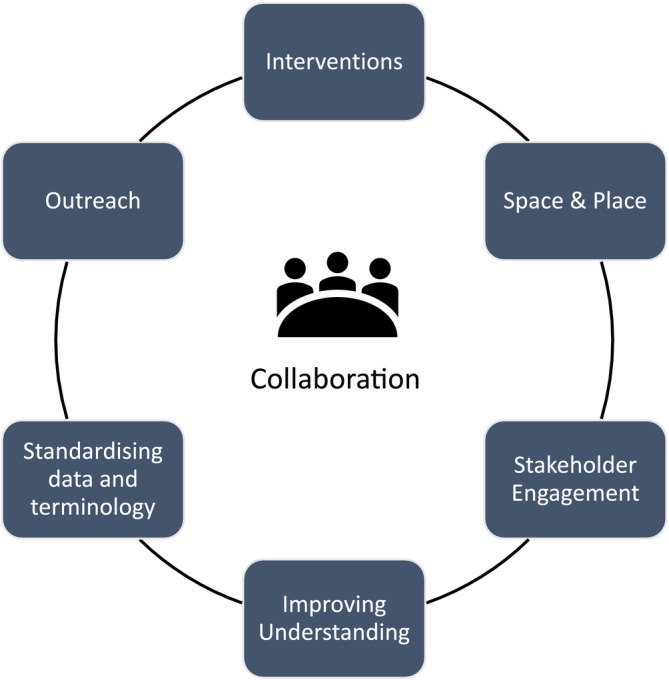
Rural mental health research categories.

**FIGURE 3:**
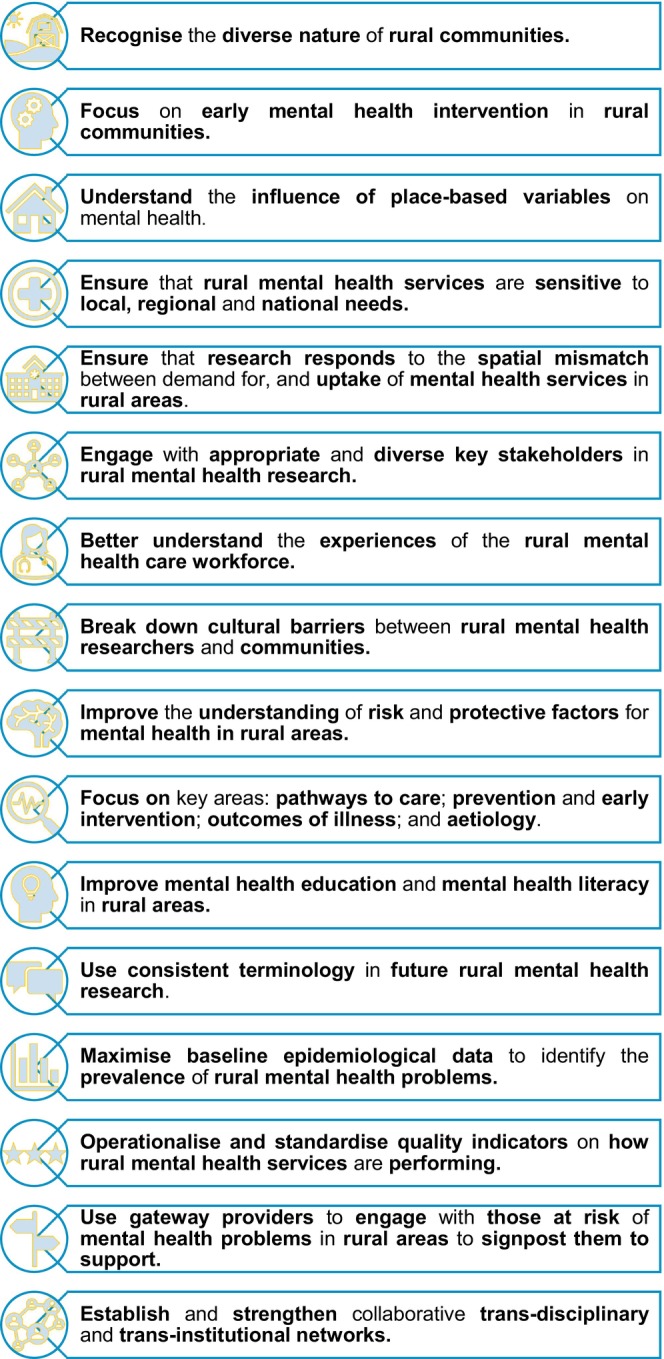
To advance rural mental health research, we need to.

Of the 20 included articles, eight were from Australia [22, 23, 24, 25, 31, 35, 36, 37], nine from the USA [28, 30, 32, 33, 34, 38, 39, 40, 41], with two from the UK [27, 29], and one having no geographical focus [26]. Papers were published between 1994 and 2025. A rapid content analysis identified seven broad categories: interventions; space and place; stakeholder engagement; improving understanding; standardising data and terminology; outreach; and collaboration. Figure 2 shows the connections between the seven broad categories. Across the seven broad categories, there were 16 priority areas (Figure 3). Across the 16 priority areas, there were 53 sub‐priorities. Priorities 1–3 were the most commonly mentioned.

The most common sub‐priority ‘Inclusion of indigenous populations and focus on their health, including mental health’, from priority 1, was mentioned in six of the 20 publications (see Supporting Information S4). Priority 2—Rural mental health research should focus on early intervention in rural communities, was mentioned in 10 of the 20 publications, which made it the most commonly cited priority. The Supporting Information S4 shows in which of the 20 publications the categories, priorities, and sub‐priorities appeared.

#### 3.2.1 Category 1—Interventions

The literature on mental health priority setting emphasised the importance of early intervention with rural communities. It was also felt that groups in rural communities at risk of mental health challenges should be prioritised. Interventions should be evidence‐based, introduced early, and targeted at those at risk of mental health challenges. Early intervention and prevention also appeared in Category 4 as areas that require improved understanding (3.2.4). To that end, participatory research can be a key gateway (3.2.3). It is also vital that those with lived experience of mental health are involved with designing such interventions, so they are responsive to their needs. This category, including the priorities and sub‐priorities, were mentioned 19 times across 13 of the 20 included publications (see Supporting Informating S4) [22, 23, 26, 27, 28, 32, 33, 34, 35, 36, 37, 38, 39].

#### 3.2.2 Category 2—Space and Place

The importance of space and place was identified as an area for rural mental health research to explore further. Geographical isolation, especially in rural areas, can heighten the risk of mental health challenges. There may be an urban–rural help in help‐seeking for those with mental health challenges. In that sense, interventions must be sensitive to context. After Interventions, Space and place was the second most commonly mentioned category, occurring 17 times across eight of the 20 included publications [22, 23, 27, 31, 33, 35, 40, 41].

#### 3.2.3 Category 3—Stakeholder Engagement

A key part of the Stakeholder engagement category is the need to engage the relevant stakeholders, for example, service users, service providers, communities, etc. Input from the rural mental health workforce is vital in this regard, as they will be an important of the desired collaboration between stakeholders (3.2.7). This links into the need for interventions to be sensitive to context, mentioned in 3.2.2. Participatory research is a way in which key stakeholders can be engaged. Through the involvement of key stakeholders, participatory research inform interventions (3.2.1). Stakeholder engagement was mentioned 14 times across 11 of the 20 included publications [22, 23, 25, 26, 30, 31, 32, 33, 34, 35, 36].

#### 3.2.4 Category 4—Improving Understanding

Research is vital to improve understanding of rural mental health challenges. The risk factors that increase their likelihood of experiencing mental health challenges, as well as the protective factors that mitigate this risk need to be understood better. Again, prevention (of illness) and early intervention was felt to be an important factor that needed to be prioritised (3.2.1). Pathways to care and trajectory of care link into the risk and protective factors for engaging with rural mental health services. Service provision was considered important, as it underpins part of the spatial mismatch in space and place (3.2.2), and service providers need to be engaged in interventions (3.2.1), stakeholder engagement (3.2.3), outreach (3.2.6), and collaboration (3.2.7). Improving understanding was mentioned category 13 times in seven of the 20 included publications [23, 24, 28, 31, 32, 33, 37].

#### 3.2.5 Category 5—Standardising Data and Terminology

The literature maintained that it is important that rural mental health research is adapted across different contexts. There are values in quality indicators, but how they are operationalised varies between different contexts. This category was mentioned six times in five of the 20 included publications [25, 26, 32, 34, 38].

#### 3.2.6 Category 6—Outreach

Linked to the issue of spatial mismatches between demand for, and availability of, rural mental health services (3.2.2) is the role of gateway outreach providers [42]. There is the problem of access to, and quality of, rural mental health services. To what extent can poor patient outcomes be attributed to providers or the patient themselves? Outreach was mentioned three times in two of the 20 included publications [34, 39].

#### 3.2.7 Category 7—Collaboration

Although not a category in its own right, it is important to recognise that collaboration cuts across many of the said six categories (Figure 2) [40].

### 3.3 Content Analysis of Mental Health Research Priorities in Rural Settings

See Figures 2 and 3.

## 4 Discussion

### 4.1 Summary of Key Findings

This rapid evidence review is the first to synthesise the international evidence on mental health research priorities in rural settings. The evidence uncovered by the rapid evidence review can be summarised into seven broad categories: interventions; spatial mismatch; the need for cultural adaptation frameworks; and collaboration.

Interventions need to be evidence‐based. The importance of prevention and early intervention was recognised when considering the types of interventions that might work [27, 33, 37]. Interventions would also need to better understand people's pathways to care and help‐seeking in rural areas [33, 37].

People living in rural areas may find it more difficult to access support than their urban counterparts. Therefore, transport infrastructure is likely to be more of a consideration for them in seeking help for mental health challenges [23, 27]. Consequently, the spatial mismatch between the demand for, and availability of, can be more pronounced [22]. One way of narrowing this spatial mismatch (3.2.2) [42], would be to upskill the workforce [33], who need to be engaged in rural mental health research.

Participatory research [34] and participant observation [36] are two ways in which the gap between the rural mental health workforce and the communities they serve can be narrowed. Gateway outreach providers [34] can narrow the spatial mismatch between demand for and availability of rural mental health services. They are required to engage with those at risk of mental health challenges. This also links into a need to better understand the risk factors that prompted people to seek help for their mental health and the protective factors that mitigate this risk (3.2.4) [28, 31, 32].

Different terminology can have variable meanings depending on context, which can be problematic. Cultural adaptation frameworks can enhance the transferability of definitions and concepts between different contexts [43]. Transferability needs to be applied across different contexts in relation to terminology, data collection, data linkage, and consequently, quality indicators. These frameworks could also improve the transferability of research to coastal communities, for whom the rapid evidence synthesis lacked information.

Collaboration cuts across all the six key categories of the rapid evidence synthesis. Interventions to address rural–urban differences in the uptake of mental health services requires collaboration (3.2.1) [40]. As does addressing the spatial mismatch between demand for, and availability of, mental health services in rural areas (3.2.2) [42]. Stakeholders need to be engaged through participatory research methods [34], which require collaboration between relevant agencies (3.2.3) [40].

Improving understanding of risk and protective factors [28, 31, 34] requires multi‐agency collaboration (3.2.4) [40]. Data collection, analysis, and linkage requires multiple agencies to collaborate [25, 32]. In standardising terminology, it is important that there is collaboration between relevant stakeholders about how best to operationalise meanings across different contexts (3.2.5) [40, 43]. Outreach to narrow the spatial mismatch between demand for, and availability of, rural mental health services requires collaboration between service users, providers, and communities (3.2.6) [42].

### 4.2 Research Context

The findings of the rapid evidence synthesis show how challenging the issue of rural mental health is across the world. These findings are strongly supported by existing research evidence. In their 2024 report, Mental Health Matters found that many people living in rural England had experienced a decline in their mental health and many had sought support for their condition. Many found it difficult to access the necessary mental health support, with transport and digital exclusion being major barriers [44]. The problem is compounded, at a service provision and commissioning level, by a lack of reliable data that dichotomises mental health use between rural and urban areas (see Supporting Information S4) [25, 26].

The issue of spatial mismatch between the demand for, and availability of, mental health services in rural areas is salient internationally (3.2.2) [23]. Using Penchansky's model of best fit between service users and providers, there appears to be disjuncture in availability, accessibility, acceptability, and appropriateness of rural mental health services and the complexity of navigating their way around the system [42]. This underscores the differential rural–urban outcomes in mental health. Mental health challenges in rural areas face geographical complexities, such as variable availability and quality of public transport [23, 27], as well as lack of trained staff providing the services [33].

It is important to engage communities and stakeholders in furthering the rural mental health research agenda. This reiterates a point made in a Public Health England report on health inequalities among older people in rural and coastal communities [45]. Often, it is the intended beneficiaries of rural mental health research that are most reluctant to participate in it, for example, rural and farming communities [27, 46]. This is perhaps due to perceived stigma about health and mental health literacy. Consequently, researchers need to build meaningful relationships with service users and providers, perhaps by adopting a more participatory approach (see Supplementary Appendix S4) [34].

Terminology can vary between contexts internationally. Different locations can interpret meanings differently. For instance, indigenous usually means Aborigines in Australia, native Americans in the USA, and First Nations groups in Canada. The cultural adaptation framework is a mechanism by which to enhance the transferability of concepts between different contexts. Bernal et al. [43] defined cultural adaptation as: ‘the systematic modification of an evidence‐based treatment (EBT) or intervention protocol to consider language, culture, and context in such a way that it is compatible with the client's cultural patterns, meanings, and values.’.

The framework would enable priorities to be adapted to the context in which the research is being undertaken. This would help to ensure that the research is sensitive to local language, culture, meanings, and values. A cultural adaptation framework could also adapt findings to coastal locations by considering some of their unique characteristics, including environmental risks and transient populations. Indeed, mental health can be a considerable problem in coastal areas, especially among young people, who can be at higher risk of having their mental illness undiagnosed [47, 48].

### 4.3 Strengths and Limitations

This rapid evidence synthesis was systematic, comprehensive, and replicable. It encompassed a broad range of international literature around rural mental health research priorities. This rapid evidence synthesis can provide a foundation of core principles to guide future work in rural mental health research for practitioners across the world.

A key limitation of the exercise was an absence of research evidence on coastal areas. Also, much of the research evidence in this area has a narrow geographical focus on Australia and North America. There is potential, however, to use a cultural adaptation framework in order to enhance the relevance to coastal communities [43].

By excluding non‐English language publications, grey literature, and unindexed regional studies, the rapid evidence review is potentially omitting useful sources of information in other languages and different types of publication. By having the strict inclusion criteria, the rapid evidence review possibly increases the risk of reporting bias.

While the inclusion of indigenous people was identified as a key priority in the review, there were limited references in the included publications around Indigenous governance, leadership, and benefit pathways. There was little mention of meaningful engagement with the Indigenous community in the research that was undertaken.

Because the timeframe to undertake the research was limited, there was no time in which to undertake a quality appraisal of the included publications. Doing so would have indicated the robustness of the publications included in the rapid evidence synthesis and could minimise bias [49].

Many improvements in healthcare provision will centre around technological advancements. A limitation of the evidence synthesis is that some of the included publications preceded the acceleration of technological advancements. This limits their relevance to current debates around improving healthcare service delivery.

### 4.4 Future Research, Policy, and Practice

Given the dearth of coastal‐related evidence in this synthesis, future work should consider the unique geospatial, socio‐economic, and seasonal challenges that coastal communities experience. These include: environmental risks and transient, seasonal populations, which can aggravate mental health challenges and necessitate bespoke responses. A cultural adaptation framework [43] may improve the transferability of some of these priorities from rural to coastal communities. There are some commonalities between rural and coastal areas, but some of the unique features of coastal communities necessitate a tailored evidence synthesis.

Some of the research priorities identified in these areas, such as the involvement and conceptualisation of ‘indigenous peoples’, appear to be context‐specific. Beyond Australia and North America, it is up to other locations to interpret what ‘indigenous peoples’ means. The term ‘regional’ also has a specific meaning in the Australian context that is not shared elsewhere. Context‐specific concepts and definitions need to be transferable to other contexts. For example, indigenous people may refer to Aborigines in Australia, Native Americans in the USA, or First Nations peoples in Canada. Cultural adaptation frameworks can improve transferability to these communities [43].

Given that the inclusion of Indigenous people was identified as a priority for rural mental health research, future policy, research, and practice should be driven by closer engagement with these communities. There are approximately 370 million Indigenous peoples living in 90 countries (see Supporting Information S1) [50]. There is much research that is conducted ‘on’, but not ‘with’, them. Addressing this imbalance would be consistent with Categories 3—Stakeholder engagement, 6—Outreach, and 7—Collaboration.

This highlights the importance of having consistent terminology as outlined in the Supporting Information S4 [32]. In attempting to standardise the priorities for rural mental health research, data collection and linkage have important roles to play if used holistically [25]. These could be the practical considerations that can usefully inform policy and practice, which aim to tackle inequalities in rural mental health provision and outcomes.

Policy and practice in rural mental health need to include both service users and providers. Involving users and providers, perhaps through participatory research [34] or co‐design (Supporting Information S4), may increase the legitimacy of such interventions. Researchers have to build meaningful relationships with key stakeholders, for example youth groups in the UK and Australian coastal areas, where mental health is a major problem, particularly for children and young people [11, 48]. Best practice should be shared, where possible. The effectiveness of the interventions needs to be evaluated over time.

## 5 Conclusion

The rapid evidence synthesis provides a useful international signpost for informing local, national and international rural mental health research priority setting exercises. Given the increased policy attention on resource‐limited coastal settings that can face considerable health and mental health challenges, we encourage researchers and policy makers to conduct mental health research priority setting exercises that are unique to this geographical context.

Standardising terminology, where possible, is important to ensure consistency of approach. Involving key stakeholders is important to ensure the legitimacy of interventions. Against that, some terms have variable meanings, such as ‘indigenous’ or ‘regional’, depending on context. Most importantly, the rapid evidence synthesis establishes a baseline core set of principles from which to support future rural mental health research.

## Author Contributions

**Viet‐Hai Phung:** writing – review and editing, writing – original draft, methodology, conceptualization, formal analysis, investigation, validation, software. **David Nelson:** writing – review and editing, methodology, conceptualization, investigation, supervision. **Ros Kane:** writing – review and editing, methodology. **Kyla Pennington:** writing – review and editing, methodology, conceptualization. **Joseph Akanuwe:** writing – review and editing, methodology. **Harriet Moore:** writing – review and editing, methodology. **Robert Dean:** writing – review and editing, methodology. **Russell Roberts:** writing – review and editing, methodology. **Derek Ward:** writing – review and editing, methodology. **Jaspreet Phull:** writing – review and editing, methodology. **Tracy McCranor:** writing – review and editing, methodology. **Colin Hopkirk:** writing – review and editing, methodology. **Jon Mansfield:** writing – review and editing, methodology. **Richard Morriss:** writing – review and editing, methodology. **Mark Gussy:** writing – review and editing, methodology. **Dave Dawson:** writing – review and editing, project administration, resources, supervision, validation, funding acquisition, investigation, conceptualization, methodology. **Nima Moghaddam:** conceptualization, investigation, funding acquisition, writing – review and editing, validation, project administration, supervision, resources, methodology.

## Funding

This work was supported by National Institute for Health and Care Research (NIHR207514).

## Disclosure

The GLiMHR project of which this rapid evidence synthesis will inform was funded by the National Institute for Health and Care Research (Ref: NIHR207514) as part of their Mental Health Research Development Award Scheme. The views expressed in this article are solely those of the author(s) and not necessarily those of the NIHR or the Department for Health and Social Care.

## Conflicts of Interest

The authors declare no conflicts of interest.

## Supporting information


**Data S1:** Supporting information.

**Data S2:** Supporting information.

**Data S3:** Supporting information.

**Data S4:** Supporting information.

## Data Availability

Data sharing not applicable to this article as no datasets were generated or analysed during the current study.

## References

[1] D. Morales , C. Barksdale , and A. Beckel‐Mitchener , “A call to action to address rural mental health disparities,” Journal of Clinical and Translational Science 4, no. 5 (2020): 463–467, 10.1017/cts.2020.42. 33244437 PMC7681156

[2] A. Edwards , R. Hung , J. Levin , et al., “Health Disparities Among Rural Individuals With Mental Health Conditions: A Systematic Literature Review,” Rural Mental Health 47, no. 3 (2023): 163–178, 10.1037/rmh0000228. 37638091 PMC10449379

[3] A. Campbell , T. Manoff , and J. Caffery , “Rurality and Mental Health: An Australian Primary Care Study,” Rural and Remote Health 6, no. 3 (2006): 1–9. 16942407

[4] P. Batterham , K. Brown , A. Trias , et al., “Systematic Review of Quantitative Studies Assessing the Relationship Between Environment and Mental Health in Rural Areas,” Australian Journal of Rural Health 30, no. 3 (2022): 306–320, 10.1111/ajr.12851. 35189016 PMC9303895

[5] W. Bird , “Improving Health in Coastal Communities,” BMJ 374 (2021): 1–2, 10.1136/bmj.n2214. 34535467

[6] S. Asthana and A. Gibson , “Averting a Public Health Crisis in England's Coastal Communities: A Call for Public Health Research and Policy,” Journal of Public Health 44, no. 3 (2021): 642–650, 10.1093/pubmed/fdab130. PMC9424058 33982058

[7] C. Whitty , “Health in Coastal Communities 2021,” https://assets.publishing.service.gov.uk/government/uploads/system/uploads/attachment_data/file/1005216/cmo‐annual_report‐2021‐health‐in‐coastal‐communities‐accessible.pdf.

[8] C. Whitty , “Health in an Ageing Society 2023,” https://assets.publishing.service.gov.uk/media/6674096b64e554df3bd0dbc6/chief‐medical‐officers‐annual‐report‐2023‐web‐accessible.pdf.

[9] C. Peng , K. Yamashita , and E. Kobayashi , “Effects of the Coastal Environment on Well‐Being,” Journal of Coastal Zone Management 19, no. 2 (2016): 421, 10.4172/2473-3350.1000421.

[10] M. White , I. Alcock , B. Wheeler , et al., “Coastal Proximity, Health and Well‐Being: Results From a Longitudinal Panel Survey,” Health & Place 23 (2013): 97–103, 10.1016/j.healthplace.2013.05.006. 23817167

[11] L. Oostenbach , J. Noall , K. Lamb , et al., “Associations Between Coastal Proximity and Children's Mental Health in Australia,” Geographical Research 61, no. 2 (2022): 248–258, 10.1111/1745-5871.12576.

[12] R. Viergever , S. Olifson , A. Ghaffar , R. F. Viergever , and R. F. Terry , “A Checklist for Health Research Priority Setting: Nine Common Themes of Good Practice,” Health Research Policy and Systems 8 (2010): 36, 10.1186/1478-4505-8-36. 21159163 PMC3018439

[13] Lincolnshire County Council , Lincolnshire Suicide Audit 2023 (Lincolnshire County Council, 2023).

[14] K. Forster , “Map Shows Areas of England Where the Most Antidepressants Are Prescribed,” The Independent (2017), https://www.independent.co.uk/news/health/antidepressants‐england‐uk‐prescribed‐most‐north‐east‐rises‐seven‐times‐prescriptions‐depression‐mental‐health‐exasol‐a7680836.html.

[15] Lincolnshire County Council , Director of Public Annual Report 2024—Integrated Care Close to Home: Creating Healthy Communities in Lincolnshire (Lincolnshire County Council, 2024).

[16] A. Tricco , J. Antony , W. Zarin , et al., “A Scoping Review of Rapid Review Methods,” BMC Medicine 13, no. 224 (2015): 1–15, 10.1186/s12916-015-0465-6. 26377409 PMC4574114

[17] D. Moher , L. Stewart , and P. Shekelle , “All in the Family: Systematic Reviews, Rapid Reviews, Scoping Reviews, Realist Reviews, and More,” Systematic Reviews 4, no. 183 (2015): 1–2, 10.1186/s13643-015-0163-7. 26693720 PMC4688988

[18] C. Garritty , G. Gartlehner , B. Nussbaumer‐Streit , et al., “Cochrane Rapid Reviews Methods Group Offers Evidence‐Informed Guidance to Conduct Rapid Reviews,” Journal of Clinical Epidemiology 130 (2021): 13–22, 10.1016/j.jclinepi.2020.10.007. 33068715 PMC7557165

[19] D. Moher , A. Liberati , J. Tetzlaff , and D. G. Altman , “Preferred Reporting Items for Systematic Reviews and Meta‐Analyses: The PRISMA Statement,” PLoS Medicine 6, no. 7 (2009): e1000097, 10.1371/journal.pmed.1000097. 19621072 PMC2707599

[20] Covidence (2024), https://www.covidence.org/.

[21] A. Tong , A. Synnot , S. Crowe , et al., “Reporting Guideline for Priority Setting of Health Research (REPRISE),” BMC Medical Research Methodology 19, no. 243 (2019): 1–11, 10.1186/s12874-019-0889-3. 31883517 PMC6935471

[22] P. Baker , D. Hegney , C. Rogers‐Clark , et al., “Planning Research in Rural and Remote Areas,” Rural and Remote Health 4, no. 2 (2004): 1–11. 15884992

[23] R. Eley and P. Baker , “Rural and Remote Health Research: Key Issues for Health Providers in Southern Queensland,” Australian Journal of Rural Health 15, no. 6 (2007): 368–372, 10.1111/j.1440-1584.2007.00918.x. 17970899

[24] M. McAllister , J. Munday , M. Taikato , B. Waterhouse , and P. K. Dunn , “Determining Mental Health Research Priorities in a Queensland Region: An Inclusive and Iterative Approach With Mental Health Service Clinicians, Consumers and Carers,” Advances in Mental Health 10, no. 3 (2012): 268–276, 10.5172/jamh.2012.10.3.268.

[25] D. Perkins , J. Farmer , L. Salvador‐Carulla , H. Dalton , and G. Luscombe , “The Orange Declaration on Rural and Remote Mental Health,” Australian Journal of Rural Health 27, no. 5 (2019): 374–379, 10.1111/ajr.12560. 31515882

[26] R. Roberts , S. Munoz , K. Thorpe , et al., “International Declaration on Rural Mental Health Research: 10 Guiding Principles and Standards,” Australian Journal of Rural Health 32, no. 4 (2024): 611–616, 10.1111/ajr.13167. 39192494

[27] B. Teague , L. Crouch‐Read , and E. Haley , “Informing Mental Health Research Priorities and Design With Rural and Agricultural Communities: A Public Involvement Consultation Case Study,” Mental Health and Social Inclusion 29 (2025): 3, 10.1108/MHSI-01-2025-0003.

[28] I. Abraham , J. Buckwalter , J. Neese , et al., “Mental Health of Rural Elderly: A Research Agenda for Nursing,” Issues in Mental Health Nursing 15, no. 3 (1994): 203–213, 10.3109/01612849409009384. 7829311

[29] C. Boyd and H. Parr , “Social Geography and Rural Mental Health Research,” Rural and Remote Health 8, no. 1 (2008): 804. 18251630

[30] E. Carpenter‐Song and C. Snell‐Rood , “The Changing Context of Rural America: A Call to Examine the Impact of Social Change on Mental Health and Mental Health Care,” Psychiatric Services 68, no. 5 (2017): 503–506, 10.1176/appi.ps.201600024. 27842467

[31] C. Fraser , F. Judd , H. Jackson , G. Murray , J. Humphreys , and G. A. Hodgins , “Does One Size Really Fit All? Why the Mental Health of Rural Australians Requires Further Research,” Australian Journal of Rural Health 10, no. 6 (2002): 288–295, 10.1046/j.1440-1584.2002.00463.x. 12472610

[32] T. Handley , K. Inder , B. Kelly , et al., “Urban–Rural Influences on Suicidality: Gaps in the Existing Literature and Recommendations for Future Research,” Australian Journal of Rural Health 19, no. 6 (2011): 279–283, 10.1111/j.1440-1584.2011.01235.x. 22098210

[33] D. Hartley , C. Britain , and S. Sulzbacher , “Behavioral Health: Setting the Rural Health Research Agenda,” Journal of Rural Health 18, no. 5 (2002): 242–255, 10.1111/j.1748-0361.2002.tb00934.x. 12061517

[34] E. Hauenstein , “Building the Rural Mental Health System: From de Facto System to Quality Care,” in Annual Review of Nursing Research, vol. 5, ed. J. Fitzpatrick and E. Merwin (Springer, 2014), 143–174. 18709749

[35] F. Hourihan and B. Kelly , “National Health Policy: What Does This Mean for Rural Mental Health Research?,” Australian Journal of Rural Health 14, no. 2 (2006): 49–50, 10.1111/j.1440-1584.2006.00762.x. 16512788

[36] F. Judd , “Progressing the Agenda for Rural Mental Health Research,” Rural and Remote Health 6, no. 3 (2006): 615. 16952272

[37] F. Judd , G. Murray , C. Fraser , J. Humphreys , G. Hodgins , and H. Jackson , “The Mental Health of Rural Australians: Developing a Framework for Strategic Research,” Australian Journal of Rural Health 10, no. 6 (2002): 296–301, 10.1046/j.1440-1584.2002.00438.x. 12472611

[38] P. Keller , D. Murray , and D. Hargrove , “A Rural Mental Health Research Agenda: Defining Context and Setting Priorities,” Journal of Rural Health 15, no. 3 (1999): 316–325, 10.1111/j.1748-0361.1999.tb00753.x. 11942564

[39] K. Rost , J. Fortney , and J. Smith , “Use, Quality, and Outcomes of Care for Mental Health: The Rural Perspective,” Medical Care Research and Review 59, no. 3 (2002): 231–265, 10.1177/1077558702059003001. 12205828

[40] L. Thorndyke , “Rural Women's Health: A Research Agenda for the Future,” Women's Health Issues 15, no. 5 (2005): 200–203, 10.1016/j.whi.2005.07.004. 16165004

[41] M. Wagenfeld , “Mental Health and Rural America: A Decade Review,” Journal of Rural Health 6, no. 4 (1990): 307–322, 10.1111/j.1748-0361.1990.tb00685.x. 10107687

[42] R. Penchansky and J. Thomas , “The Concept of Access: Definition and Relationship to Consumer Satisfaction,” Medical Care 19, no. 2 (1981): 127–140, 10.1097/00005650-198102000-00001. 7206846

[43] G. Bernal , M. Jimenez‐Chafey , and M. Domenech‐Rodriguez , “Cultural Adaptation of Treatments: A Resource for Considering Culture in Evidence‐Based Practice,” Professional Psychology: Research and Practice 40, no. 4 (2009): 361–368, 10.1037/a0016401.

[44] Mental Health Matters. Insight Into the Mental Health of Rural Communities (Mental Health Matters, 2024).

[45] Public Health England , An Evidence Summary of Health Inequalities in Older Populations in Coastal and Rural Areas (Public Health England, 2019).

[46] H. Bird , M. Gussy , D. Nelson , et al., “Toolkit for Increasing the Participation of Rural and Coastal Communities in Health and Social Care Research,” (2024), https://bpb‐eu‐w2.wpmucdn.com/blogs.lincoln.ac.uk/dist/6/8425/files/2024/04/Rural‐and‐Coastal‐Health‐and‐Care‐Research‐Toolkit‐5df5831512562fb8.pdf.

[47] C. Wicks , S. McPherson , C. Booker , A. Trotta , M. Kumari , and E. T. Murray , “Risk of Diagnosed and Undiagnosed Mental Distress in Coastal and Inland English Residents: A Pooled Cross‐Sectional Analysis of Adult UKHLS Respondents,” Health & Place 95 (2025): 3501, 10.1016/j.healthplace.2025.103501. 40633447

[48] E. Murray and C. Wicks , Coastal Disadvantage and Youth Mental Health: Emerging Evidence From England (University of Essex Centre for Coastal Communities, 2023).

[49] Joanna Briggs Institute , “Critical Appraisal Tools,” (2024), https://jbi.global/critical‐appraisal‐tools.

[50] S. Harfield , O. Pearson , K. Morey , et al., “Assessing the Quality of Health Research From an Indigenous Perspective: The Aboriginal and Torres Strait Islander Quality Appraisal Tool,” BMC Medical Research Methodology 20, no. 79 (2020): 1–9, 10.1186/s12874-020-00959-3. PMC7147059 32276606

