# Supplementary material for: Identifying the Mental Health Research Priorities in Rural Settings, With Implications for Coastal Communities: A Rapid Evidence Synthesis
Source: Aust J Rural Health. 2026 Mar 20;34(2):e70171. doi: 10.1111/ajr.70171 (PMC13003580; doi:10.1111/ajr.70171)
Supplement: Supplementary file 1 — Data S1: Supporting information. [file AJR-34-0-s004.pdf]

ABORIGINAL AND TORRES STRAIT ISLANDER QUALITY APPRAISAL TOOL

Answer either “Yes”, “Partially”, “No” or “Unclear” to each question

Article citation: ____ Date: 09 May 2025

Reviewer’s name: ____

Question	Yes	Partially	No	Unclear
1. Did the research respond to a need or priority determined by the community?	☐	☐	X	☐
2. Was community consultation and engagement appropriately inclusive?	☐	☐	X	☐
3. Did the research have Aboriginal and Torres Strait Islander research leadership?	☐	☐	X	☐
4. Did the research have Aboriginal and Torres Strait Islander governance?	☐	☐	X	☐
5. Were local community protocols respected and followed?	☐	☐	X	☐
6. Did the researchers negotiate agreements in regards to rights of access to Aboriginal and Torres Strait Islander peoples’ <u>existing</u> intellectual and cultural property?	☐	☐	X	☐
7. Did the researchers negotiate agreements to protect Aboriginal and Torres Strait Islander peoples’ ownership of intellectual and cultural property <u>created</u> through the research?	☐	☐	X	☐
8. Did Aboriginal and Torres Strait Islander peoples and communities have control over the collection and management of research materials?	☐	☐	X	☐
9. Was the research guided by an Indigenous research paradigm?	☐	☐	X	☐
10. Does the research take a strengths-based approach, acknowledging and moving beyond practices that have harmed Aboriginal and Torres Strait peoples in the past?	☐	☐	X	☐
11. Did the researchers plan and translate the findings into sustainable changes in policy and/or practice?	☐	☐	X	☐
12. Did the research benefit the participants and Aboriginal and Torres Strait Islander communities?	☐	☐	X	☐

13. Did the research demonstrate capacity strengthening for Aboriginal and Torres Strait Islander individuals?	☐	☐	X	☐
14. Did everyone involved in the research have opportunities to learn from each other?	☐	☐	X	☐
