# Supplementary material for: Identifying the Mental Health Research Priorities in Rural Settings, With Implications for Coastal Communities: A Rapid Evidence Synthesis
Source: Aust J Rural Health. 2026 Mar 20;34(2):e70171. doi: 10.1111/ajr.70171 (PMC13003580; doi:10.1111/ajr.70171)
Supplement: Supplementary file 3 — Data S3: Supporting information. [file AJR-34-0-s003.docx]

# REPRISE Framework

| **o** | **Item** | **Descriptor and/or examples** |
| --- | --- | --- |
| **A** | **Context and scope** | |
| 1 | Define geographical scope | 20 included publications - 8 from Australia(1-8), 9 from the USA(9-17), 2 from the UK(18, 19), 1 with no geographical scope(20). Six of the 20 included publications used priority setting to establish priorities for rural mental health research.(1, 2, 7, 8, 19, 20) |
| 2 | Define health area, field, focus | Rural mental health generally. Some publications focused on specific sub-populations, such as the elderly(9), women(16) One publications focused on health research in rural areas more generally.(2) One publication focused on specific matters, linked to mental health – suicide.(11) |
| 3 | Define the intended beneficiaries | The Baker (2004) study aimed to improve the rural medical workforce.(1)  To determine what community health service providers in rural southern Queensland considered were major issues affecting their efficacy. Results will inform the future research strategy of the Centre for Rural and Remote Area Health with the aim of addressing specific regional needs.(2)  The following research aims were identified:  1. To identify and prioritise research topics of importance to the Mental Health Service (MHS) of the Sunshine Coast Health Service District (SCHSD);  2. To compare local priorities with national and state research agendas.(7)  As a group, we take this declaration as an opportunity to invite discussion about how we can collectively improve the mental health of rural residents through research, service design and delivery.(8)  Rural communities have unique mental health needs and challenges which are often related to the uniqueness of the community itself. On a per- capita basis, the investment in rural mental health research is far less than that in urban communities. Added to this, rural communities are often at risk of researchers, based in large urban universities, visiting, conducting the research with minimal engagement with local stakeholders and limited understanding of the community's social- service- environmental context.(20)  The purpose of this case study is to undertake a public involvement consultation with members of a rural and agricultural mental health support organisation to explore mental health research priorities and factors relating to research study design that may support or prevent participation.(19) |
| 4 | Define the target audience of the priorities | The rural medical workforce.(1)  Health providers and other key stakeholders.(2)  The following research aims were identified:  1. To identify and prioritise research topics of importance to the Mental Health Service (MHS) of the Sunshine Coast Health Service District (SCHSD);  2. To compare local priorities with national and state research agendas.(7)  A call to action for communities, service providers and researchers.(8)  The aim of this commentary is to provide a reference on research practice for novice and experienced researchers on rural mental health research and practice, to assist policymakers, government and funding bodies to establish appropriate standards and guidelines for rural mental health research, and support rural communities to advocate for equity of funding and sustainable research as they engage with researchers, funders and governments.(20)  The core aim of the PPI consultation case study was to engage rural and agricultural community members of a rural mental health VCSE organisation to share their views about local priorities for mental health research affecting their community, and to discuss perceptions on study design and promotional aspects of research studies which could influence their choice to participate.(19) |
| 5 | Identify the research area | Rural mental health. |
| 6 | Identify the type of research questions | For the Baker study(1), the types of research questions discussed were:   - Health professional support and development (52 participants, 5 sub-themes). - Mechanisms for identifying local and regional needs (51 participants, 3 sub-themes). - Mental health (48 participants, 6 sub-themes). - Health and interaction with the environment (44 participants, 7 sub-themes). - Management of conditions for which little is known (34 participants, 3 sub-themes). - Post-acute care (29 participants, 6 sub-themes). - Evidence-based practice (27 participants, 2 sub-themes). - Health workforce, including volunteers (25 participants, 4 sub-themes). - Indigenous health (24 participants, 2 sub-themes). - Access to health service delivery (11 participants, split int 2 sub-themes). - Economic impact of new programmes (4 participants, 1 sub-theme). - Outcomes impact of research partnerships (3 participants, 1 sub-theme).   For the Eley (2007) study(2), 17 themes were generated:   - Workforce - Mental health - Access - Perceptions / expectations - Inter-agency co-operation - Aged care - Transport - Substance abuse - Carers - Counselling - Health education - Health information - Youth - Health providers - Disability - Chronic disease - Indigenous health.   By focusing more on process than in depth exploration of perceptions relating to research ideas, the research focused on consensus building, rather than on honing research ideas into researchable questions.(7)  Ten problems related to current models of rural mental health and well‐being were identified. They are as follows:   - Rural communities are different from cities and are not homogenous; - The rural mental health system is not working; - Top‐down service models are based on urban assumptions; - Services are not based on needs; - The current forms of public financing are misaligned; - Fragmentation and competition hinder sustainable, robust service provision; - Structural inequity in mental health service provision is amplified in rural areas; - The rural mental health workforce cannot be a miniature version of that found in large cities; - While telehealth and online services should augment mental health services for all clients whether rural or urban, people with mental health challenges often need to speak in person with a health professional, and on some occasions, very quickly; and - Data sets are incomplete, disjointed and limited: many different and incompatible data sets are gathered and there is little data‐sharing or linkage.(8)   Ten solutions are proposed for rural mental health and wellbeing that together would benefit from robust testing and evaluation. They are as follows:   - Whole‐of‐community, place‐based approaches are promising; - New service models tailored to context must be considered; - Co‐designed bottom‐up processes should be pursued in collaboration with state and federal partners; - Holistic and integrated care models need testing; - New better‐aligned funding models are needed; - Whole of community approaches are needed, not pilot studies; - New rural workforce models are needed; - Digital technology contributes now and can do more as part of new systems; and - Enhance data collection, monitoring, linkage, analysis and planning.(8)   Ten principles to guide and support mental health research with rural communities.   - Equitable rural research funding; - Equality of status for all research stakeholders; - Co- creation, co- design, co- production and co-publication; - Continuing partnership with community; - Connection to the community; - Flexibility to respond; - Confidentiality and privacy; - Data sovereignty; - Evidence- informed knowledge to action; and - Enduring positive legacy and capacity building.(20)   A short bespoke online survey (n = 29) was sent to all members of the VCSE incorporating a mix of selection and open comment questions relating to mental health research opportunity awareness, types of health studies of interest, key areas of research need, ideas to engage rural and agricultural communities in research and personal factors which encourage or prevent participation in research studies. Two in-person community consultations (n ¼ 10) were conducted to further discuss research priorities for the local rural and farming community, identification of barriers to engagement and ideas to engage farmers and their families in research studies.  Rural and agricultural research priority areas  Priority area 1: improving rural health services and care provision.  1. evaluating brief psychological therapy provisions for farmers and delivered at home or locally;  2. improving the integration of rural health services to be more flexible, responsive and collaborative; and  3. supporting health-care professionals working in rural and agricultural areas to obtain specialised training to communicate and work successfully within these communities.  Priority area 2: understanding the impact of rural living and working in mental health.  Priority area 3: strategies to improve rural community connectedness.  1. How clearly the purpose/intention of the research was explained.  2. The location of visits, and if the participant needed to travel.  3. The expected time needed to take part in the study.(19) |
| 7 | Define the time frame | The 20 included publications covered the date range from 1994-2025. |
| **B** | **Governance and team** | |
| 8 | Describe the selection and structure of the leadership and management team |  |
| 9 | Describe the characteristics of the team | Mental health researchers and service providers from New South Wales, Victoria, the Australian Capital Territory and Western Australia met in Orange in NSW in October 2018 to examine the issue of rural mental health and well‐being. A collaborative team was formed comprising multi-disciplinary experts from the University and the MHS and regular meetings were commenced in 2008 to discuss interests and concerns. The team agreed that it was important to establish local research priorities to avoid the tendency for activities to be ad hoc and to ensure that projects contribute to national and state agendas.(8)  This commentary is based on the insights of a panel of authors from 9 countries, each with extensive experience of rural mental health research and work. In addition to research and service delivery, these experiences encompass activities such as consultations, workshops, focus groups, conferences, community meetings and producing publications, toolkits and reports conducted in the course of their work.(20)  This PPI research consultation case study was conducted in a large Voluntary, Community and Social Enterprise (VCSE) organisation in the East of England. The East of England has a higher proportion of the population living in rural areas (25%–50%) compared to the UK average (ONS, 2017) and is home to 12% of all farmers in England (UK Gov, 2024b). The VCSE engages those living and working in rural and agricultural communities to support mental health concerns, including offering a helpline and funded counselling, training community volunteers and conducting community engagement and mental health awareness events. The researchers, from rural NHS and academic backgrounds, and the VCSE partner, have been working together for the past year on a growing programme of regional mental health research in rural and agricultural communities.(19) |
| 10 | Describe any training or experience relevant to conducting priority setting |  |
| **C** | **Framework for priority setting** | |
| 11 | State the framework used (if any) |  |
| **D** | **Stakeholders or participants** | |
| 12 | Define the inclusion criteria for stakeholders involved in priority-setting | In one study, consumer, academic, and health professional organisations were invited to a workshop. There were 75 participants in the workshop representing 20 different organisations. Majority of participants were health professionals from community and residential care. Other organisations represented included: police, local city council, local GPs, and psychologists.(1) The purpose of the study was to improve recruitment and retention in the rural medical workforce.  In another study, participants from organisations directly involved with health care were complemented by representatives from local government, the police service and church groups were included in a consensus workshop to consider what were major issues affecting the efficacy of provision of rural community health services. Participants from organisations directly involved with health care were complemented by representatives from local government, the police service and church groups.(2)  Another study involved a series of meetings between three distinct focus groups involving mental health service (MHS) clinicians, MHS consumers (persons who are or have accessed the mental health service) and carers of MHS consumers. The anticipated sample size at the commencement of the study was 120 participants, comprising 50 clinicians, 50 consumers and 20 carers. Overall, the research focus groups included 47 clinicians, 49 consumers and 21 carers, totalling 117 participants. The MHS (SCHSD) employs approximately 350 clinicians and as a result the research sample for this cohort was 13% of the total staff population. The approximate number of consumers opened to the Service at any point in time is 1200 people which means the research sample group was 4% of the total population.(7)  In another study, mental health researchers and service providers from New South Wales, Victoria, Western Australia and the Australian Capital Territory met in Orange in October 2018 to explore issues pertaining to rural mental health and well‐being.(8)  One paper describes how an international panel of rural mental health leaders adapted digital conferencing and the rapid synthesis and translation process (RSTP) to build consensus on core principles of rural mental health research and their application to policy and practice. This declaration statement draws the authors' extensive collective knowledge built over many years of working with rural communities across nine countries. In addition to research and service delivery, these experiences encompass activities such as consultations, workshops, focus groups, conferences, community meetings and producing publications, toolkits and reports conducted in the course of their work.(20)  There was also a public involvement consultation with members of a rural and agricultural mental health support organisation to explore mental health research priorities and factors relating to research study design that may support or prevent participation.(19) |
| 13 | State the strategy or method for identifying and engaging stakeholders | In the Baker et al. study (2004), all participating organisations had been sent a letter inviting them to send at least one representative to attend. These organisations represented consumers, academics, and health professionals. The purpose of the study was to improve recruitment and retention in the rural medical workforce.(1)  Key stakeholders in health provision and other organisations with an interest in community health in the target communities were invited. Participants were recruited through existing networks of Centre for Rural and Remote Area Health (CRRAH) and through local radio and newspapers. In addition, snowball sampling (Morrison, 1988) was utilised, where prospective participants were asked about other stakeholders who might wish to attend the workshop.(2)  A purposive sampling strategy was used to target relevant consumer and carer groups. In this study the experts were considered to be consumers, carers and clinicians from the region. Consumer confidentiality disallowed direct approaches to known consumers of the Service.(7)  Over 2 days, mental health researchers, academics, service providers, managers and commissioners identified problems and solutions: making use of national and international experience and evidence about service models, evidence from small‐scale local pilots and novel data analyses.(8)  This declaration statement draws the authors' extensive collective knowledge built over many years of working with rural communities across nine countries. In addition to research and service delivery, these experiences encompass activities such as consultations, workshops, focus groups, conferences, community meetings and producing publications, toolkits and reports conducted in the course of their work.(20)  The core aim of the PPI consultation case study was to engage rural and agricultural community members of a rural mental health VCSE organisation to share their views about local priorities for mental health research affecting their community, and to discuss perceptions on study design and promotional aspects of research studies which could influence their choice to participate. A short bespoke online survey (n = 29) was sent to all members of the VCSE incorporating a mix of selection and open comment questions relating to mental health research opportunity awareness, types of health studies of interest, key areas of research need, ideas to engage rural and agricultural communities in research and personal factors which encourage or prevent participation in research studies. This PPI research consultation case study was conducted in a large Voluntary, Community and Social Enterprise (VCSE) organisation in the East of England. The East of England has a higher proportion of the population living in rural areas (25%–50%) compared to the UK average (ONS, 2017) and is home to 12% of all farmers in England (UK Gov, 2024b). The VCSE engages those living and working in rural and agricultural communities to support mental health concerns, including offering a helpline and funded counselling, training community volunteers and conducting community engagement and mental health awareness events. The researchers, from rural NHS and academic backgrounds, and the VCSE partner, have been working together for the past year on a growing programme of regional mental health research in rural and agricultural communities.(19) |
| 14 | Indicate the number of participants and/or organizations involved | There were 75 participants representing 20 different organisations. 12 were from nursing / psychology at the University of Southern Queensland, 11 from the Queensland Department of Health, 6 represented aged care facilities / aged care community providers.(1)  A total of 85 participants representing 47 services and 41 different organisations attended the eight workshops. Represented were national agencies (5), care delivery organisations (8), community health services (9), local government (2), counselling services (3), private and public hospitals (6), Indigenous medical services (2), pastoral services (5), health professional support organisations (4) and tertiary educational establishments (3).(2)  Overall, the research focus groups included 47 clinicians, 49 consumers and 21 carers, totalling 117 participants.(7)  Over 2 days, mental health researchers, academics, service providers, managers and commissioners identified problems and solutions: making use of national and international experience and evidence about service models, evidence from small‐scale local pilots and novel data analyses. Further teleconferences and five iterations of the draft statement were considered before reaching a final consensus. There was a strong consensus around the problems and potential solutions that would benefit from being examined robustly.(8)  This paper describes how an international panel of rural mental health leaders adapted digital conferencing and the rapid synthesis and translation process (RSTP) to build consensus on core principles of rural mental health research and their application to policy and practice. This declaration statement draws the authors' extensive collective knowledge built over many years of working with rural communities across nine countries.(20)  The age of the 39 PPI participants ranged from 19 to over 80years. Almost half (47%) of survey respondents directly worked in the local farming and agricultural industry at the time of data collection.(19) |
| 15 | Describe the characteristics of stakeholders | There were 75 participants in the workshop representing 20 different organisations. Majority of participants were health professionals from community and residential care. Other organisations represented included: police, local city council, local GPs, and psychologists.(1)  In another study, participants from organisations directly involved with health care were complemented by representatives from local government, the police service and church groups were included in a consensus workshop to consider what were major issues affecting the efficacy of provision of rural community health services. Participants from organisations directly involved with health care were complemented by representatives from local government, the police service and church groups.(2)  Overall, the research focus groups included 47 clinicians, 49 consumers and 21 carers, totalling 117 participants.(7)  Over 2 days, mental health researchers, academics, service providers, managers and commissioners identified problems and solutions: making use of national and international experience and evidence about service models, evidence from small‐scale local pilots and novel data analyses. Further teleconferences and five iterations of the draft statement were considered before reaching a final consensus. There was a strong consensus around the problems and potential solutions that would benefit from being examined robustly.(8)  This paper describes how an international panel of rural mental health leaders adapted digital conferencing and the rapid synthesis and translation process (RSTP) to build consensus on core principles of rural mental health research and their application to policy and practice. This declaration statement draws the authors' extensive collective knowledge built over many years of working with rural communities across nine countries.(20)  The age of the 39 PPI participants ranged from 19 to over 80years. Almost half (47%) of survey respondents directly worked in the local farming and agricultural industry at the time of data collection.(19) |
| 16 | State if reimbursement for participation was provided | N/A |
| **E** | **Identification and collection of research priorities** | |
| 17 | Describe methods for collecting initial priorities | NGT was used with a facilitator and four note-takers. Allowed all participants to have equal say. After all participants had equal say, facilitators analysed ideas thematically. Then, all participants ranked the themes by importance.(1)  The workshops used the nominal group technique to identify what participants considered were key health issues in their locations.(2)  A total of 29 focus groups were held over a 6 month period. This included 18 focus groups for clinicians, four focus groups for carers and seven focus groups for consumers. The size of the focus groups varied from 17 participants to three. Consumer and carer groups tended to be larger due to participants being accessed during a pre-existing community forum. A special focus group for Indigenous mental health consumers (N = 7), representing 14% of the consumer cohort population, was commenced in the second round to enable a voice for this minority group. Over the life of the study, a total of 230 individual responses were received across three rounds.(7)  Over 2 days, mental health researchers, academics, service providers, managers and commissioners identified problems and solutions: making use of national and international experience and evidence about service models, evidence from small‐scale local pilots and novel data analyses. Further teleconferences and five iterations of the draft statement were considered before reaching a final consensus which is shared by those listed below. There was a strong consensus around the problems and potential solutions that would benefit from being examined robustly.(8)  This paper describes how an international panel of rural mental health leaders adapted digital conferencing and the rapid synthesis and translation process (RSTP) to build consensus on core principles of rural mental health research and their application to policy and practice. This declaration statement draws the authors' extensive collective knowledge built over many years of working with rural communities across nine countries. In addition to research and service delivery, these experiences encompass activities such as consultations, workshops, focus groups, conferences, community meetings and producing publications, toolkits and reports conducted in the course of their work. The digital conferencing and RSTP provided a mechanism for the authors to offer a formal account of their experience and insights related to rural mental health research. The authors included mental health consumers, carers, researchers, clinicians, service managers and policy analysts working in diverse social, cultural and environmental contexts across the globe. The collective insights and themes were collated and distilled into core themes using expert- based consensus through digital conferencing. The themes were then shaped and rewritten once consensus was agreed via asynchronous communication.(20)  A short bespoke online survey (n = 29) was sent to all members of the VCSE incorporating a mix of selection and open comment questions relating to mental health research opportunity awareness, types of health studies of interest, key areas of research need, ideas to engage rural and agricultural communities in research and personal factors which encourage or prevent participation in research studies.(19) |
| 18 | Describe methods for collating and categorizing priorities | NGT was used to collate and categorise priorities. A facilitator collected ideas from each of the group. NGT allowed each of the group equal say. Ideas then analysed thematically by facilitators. Then all group members ranked ideas by order of importance.(1)  The workshops used the nominal group technique to identify key health issues (O’Neil & Jackson, 1983). The workshop began with silent generation onto cards of key health issues. Ideas from the participants were then presented in plenary until all issues had been exhausted. Issues were then pooled into themes and participants were given the opportunity to rank the key issues. Those with the highest priority were discussed further in small groups and key points presented.(2)  A modified Delphi method was identified as the most appropriate research design. In this study the experts were considered to be consumers, carers and clinicians from the region. The funnelling approach was achieved through using three rounds of consultations with these experts. Qualitative data analysis involved simple content analysis. That is, research ideas were grouped into themes by two members of the team independently and findings discussed with the research team. Data from each cohort were distilled after each round and feedback on emerging findings were collated and distributed to the cohorts in subsequent rounds until consensus was reached. The team also reflected on the process occurring at each round, identifying impediments to, and strategies for, sustaining participation from the experts. By focusing more on process than in depth exploration of perceptions relating to research ideas, the research focused on consensus building, rather than on honing research ideas into researchable questions.(7)  Over 2 days, mental health researchers, academics, service providers, managers and commissioners identified problems and solutions: making use of national and international experience and evidence about service models, evidence from small‐scale local pilots and novel data analyses. Further teleconferences and five iterations of the draft statement were considered before reaching a final consensus which is shared by those listed below. There was a strong consensus around the problems and potential solutions that would benefit from being examined robustly.(8)  The digital conferencing and rapid synthesis and translation process (RSTP) provided a mechanism for the authors to offer a formal account of their experience and insights related to rural mental health research. The authors included mental health consumers, carers, researchers, clinicians, service managers and policy analysts working in diverse social, cultural and environmental contexts13 across the globe. The collective insights and themes were collated and distilled into core themes using expert- based consensus through digital conferencing. The themes were then shaped and rewritten once consensus was agreed via asynchronous communication.(20)  Survey data were collected online via MS Forms promoted through the VSCE website and email distribution list. Community discussions were not recorded to preserve anonymity within a wider group environment, but extensive contemporaneous notes were taken by the researcher at the point of discussion and enhanced by further reflections between the authors who were all present at one of the community discussions, but themselves did not contribute data to those sessions. Survey data relating to preferences and awareness are presented descriptively using frequencies and proportions. Open comment boxes were analysed using summative content analysis (Graneheim and Lundman, 2004), examining the most frequently responses relating to priorities and views. Line-by-line open coding of survey comment box responses was performed, contextualised with insights and quotes manually written from community discussions and resulting initial codes clustered into initial themes relating to research priorities and preferences in mental health research design.(19) |
| 19 | Describe methods and reasons for modifying (removing, adding, reframing) priorities | NGT was used to collate and categorise priorities. A facilitator collected ideas from each of the group. NGT allowed each of the group equal say. Ideas then analysed thematically by facilitators. Then all group members ranked ideas by order of importance.(1)  The workshops used the nominal group technique to identify key health issues (O’Neil & Jackson, 1983). The workshop began with silent generation onto cards of key health issues. Ideas from the participants were then presented in plenary until all issues had been exhausted. Issues were then pooled into themes and participants were given the opportunity to rank the key issues. Those with the highest priority were discussed further in small groups and key points presented.(2)  Data from each cohort were distilled after each round and feedback on emerging findings were collated and distributed to the cohorts in subsequent rounds until consensus was reached (Greatorex & Dexter, 2000). This method allows for clarification of any agreement that exists amongst the group and is a standard approach to identifying priorities in a Delphi method (Kirkwood, Wales, & Wilson, 2003). The team also reflected on the process occurring at each round, identifying impediments to, and strategies for, sustaining participation from the experts. For example, during round two, it became clear that the indigenous arm of the service was not engaged. On the advice of Indigenous mental health workers, a more culturally- appropriate version of the information provided at each round was constructed and used for all groups. By focusing more on process than in depth exploration of perceptions relating to research ideas, the research focused on consensus building, rather than on honing research ideas into researchable questions.(7)  Over 2 days, mental health researchers, academics, service providers, managers and commissioners identified problems and solutions: making use of national and international experience and evidence about service models, evidence from small‐scale local pilots and novel data analyses. Further teleconferences and five iterations of the draft statement were considered before reaching a final consensus. There was a strong consensus around the problems and potential solutions that would benefit from being examined robustly.(8)  In addition to research and service delivery, these experiences encompass activities such as consultations, workshops, focus groups, conferences, community meetings and producing publications, toolkits and reports conducted in the course of their work. The digital conferencing and RSTP provided a mechanism for the authors to offer a formal account of their experience and insights related to rural mental health research. The authors included mental health consumers, carers, researchers, clinicians, service managers and policy analysts working in diverse social, cultural and environmental contexts13 across the globe. The collective insights and themes were collated and distilled into core themes using expert- based consensus through digital conferencing. The themes were then shaped and rewritten once consensus was agreed via asynchronous communication.  Ten principles to guide and support mental health research with rural communities.   - Equitable rural research funding; - Equality of status for all research stakeholders; - Co- creation, co- design, co- production and co-publication; - Continuing partnership with community; - Connection to the community; - Flexibility to respond; - Confidentiality and privacy; - Data sovereignty; - Evidence- informed knowledge to action; and - Enduring positive legacy and capacity building.(20)   A short bespoke online survey (n = 29) was sent to all members of the VCSE incorporating a mix of selection and open comment questions relating to mental health research opportunity awareness, types of health studies of interest, key areas of research need, ideas to engage rural and agricultural communities in research and personal factors which encourage or prevent participation in research studies. Two in-person community consultations (n ¼ 10) were conducted to further discuss research priorities for the local rural and farming community, identification of barriers to engagement and ideas to engage farmers and their families in research studies.(19) |
| 20 | Describe methods for refining or translating priorities into research topics or questions | NGT was used to collate and categorise priorities. A facilitator collected ideas from each of the group. NGT allowed each of the group equal say. Ideas then analysed thematically by facilitators. Then all group members ranked ideas by order of importance.(1) For the Baker study(1), in the brainstorming session, 12 themes, within which there numerous sub-themes were identified:   - Health professional support and development (52 participants, 5 sub-themes). - Mechanisms for identifying local and regional needs (51 participants, 3 sub-themes). - Mental health (48 participants, 6 sub-themes). - Health and interaction with the environment (44 participants, 7 sub-themes). - Management of conditions for which little is known (34 participants, 3 sub-themes). - Post-acute care (29 participants, 6 sub-themes). - Evidence-based practice (27 participants, 2 sub-themes). - Health workforce, including volunteers (25 participants, 4 sub-themes). - Indigenous health (24 participants, 2 sub-themes). - Access to health service delivery (11 participants, split int 2 sub-themes). - Economic impact of new programmes (4 participants, 1 sub-theme). - Outcomes impact of research partnerships (3 participants, 1 sub-theme).   Data were analysed by content analysis; thematic coding, comparison through the process of indexing and re-analysis through text search. In addition the data were quantified within and among both workshop and thematic issue according to prioritisation by participants, frequency of theme appearing and number of comments generated. Combining all these data generated a ranking of themes by importance.(2)  Over 2 days, mental health researchers, academics, service providers, managers and commissioners identified problems and solutions: making use of national and international experience and evidence about service models, evidence from small‐scale local pilots and novel data analyses. Further teleconferences and five iterations of the draft statement were considered before reaching a final consensus. There was a strong consensus around the problems and potential solutions that would benefit from being examined robustly.(8)  In addition to research and service delivery, these experiences encompass activities such as consultations, workshops, focus groups, conferences, community meetings and producing publications, toolkits and reports conducted in the course of their work. The digital conferencing and RSTP provided a mechanism for the authors to offer a formal account of their experience and insights related to rural mental health research. The authors included mental health consumers, carers, researchers, clinicians, service managers and policy analysts working in diverse social, cultural and environmental contexts13 across the globe. The collective insights and themes were collated and distilled into core themes using expert- based consensus through digital conferencing. The themes were then shaped and rewritten once consensus was agreed via asynchronous communication.  Ten principles to guide and support mental health research with rural communities.   - Equitable rural research funding; - Equality of status for all research stakeholders; - Co- creation, co- design, co- production and co-publication; - Continuing partnership with community; - Connection to the community; - Flexibility to respond; - Confidentiality and privacy; - Data sovereignty; - Evidence- informed knowledge to action; and - Enduring positive legacy and capacity building.(20)   A short bespoke online survey (n = 29) was sent to all members of the VCSE incorporating a mix of selection and open comment questions relating to mental health research opportunity awareness, types of health studies of interest, key areas of research need, ideas to engage rural and agricultural communities in research and personal factors which encourage or prevent participation in research studies. Two in-person community consultations (n ¼ 10) were conducted to further discuss research priorities for the local rural and farming community, identification of barriers to engagement and ideas to engage farmers and their families in research studies.(19) |
| 21 | Describe methods for checking whether research questions or topics have been answered | NGT was used to collate and categorise priorities. A facilitator collected ideas from each of the group. NGT allowed each of the group equal say. Ideas then analysed thematically by facilitators. Then all group members ranked ideas by order of importance.(1)  Data were analysed by content analysis; thematic coding, comparison through the process of indexing and re-analysis through text search. In addition the data were quantified within and among both workshop and thematic issue according to prioritisation by participants, frequency of theme appearing and number of comments generated. Combining all these data generated a ranking of themes by importance.(2)  Figure 1 shows the three rounds of focus group meetings, each with their own purpose: idea generation, priority setting and choosing the top three themes for future research. For each round the research tool changed, commencing with an open question – What are the top priority research ideas you have for the Sunshine Coast Health Service District Mental Health Service? Participants in the second round of focus groups were asked to identify their most important ideas from a list of 16 ideas generated after the first round. The final round involved a shorter list of 10 items, from which participants were to identify their top three topics, which we termed themes.(7)  Over 2 days, mental health researchers, academics, service providers, managers and commissioners identified problems and solutions: making use of national and international experience and evidence about service models, evidence from small‐scale local pilots and novel data analyses. Further teleconferences and five iterations of the draft statement were considered before reaching a final consensus. There was a strong consensus around the problems and potential solutions that would benefit from being examined robustly.(8)  In addition to research and service delivery, these experiences encompass activities such as consultations, workshops, focus groups, conferences, community meetings and producing publications, toolkits and reports conducted in the course of their work. The digital conferencing and RSTP provided a mechanism for the authors to offer a formal account of their experience and insights related to rural mental health research. The authors included mental health consumers, carers, researchers, clinicians, service managers and policy analysts working in diverse social, cultural and environmental contexts13 across the globe. The collective insights and themes were collated and distilled into core themes using expert- based consensus through digital conferencing. The themes were then shaped and rewritten once consensus was agreed via asynchronous communication.  Ten principles to guide and support mental health research with rural communities.   - Equitable rural research funding; - Equality of status for all research stakeholders; - Co- creation, co- design, co- production and co-publication; - Continuing partnership with community; - Connection to the community; - Flexibility to respond; - Confidentiality and privacy; - Data sovereignty; - Evidence- informed knowledge to action; and - Enduring positive legacy and capacity building.(20)   Survey data were collected online via MS Forms © promoted through the VSCE website and email distribution list. Community discussions were not recorded to preserve anonymity within a wider group environment, but extensive contemporaneous notes were taken by the researcher at the point of discussion and enhanced by further reflections between the authors who were all present at one of the community discussions, but themselves did not contribute data to those sessions.(19) |
| 22 | Describe number of research questions or topics | For the Baker study(1), in the brainstorming session, 12 themes, within which there numerous sub-themes were identified:   - Health professional support and development (52 participants, 5 sub-themes). - Mechanisms for identifying local and regional needs (51 participants, 3 sub-themes). - Mental health (48 participants, 6 sub-themes). - Health and interaction with the environment (44 participants, 7 sub-themes). - Management of conditions for which little is known (34 participants, 3 sub-themes). - Post-acute care (29 participants, 6 sub-themes). - Evidence-based practice (27 participants, 2 sub-themes). - Health workforce, including volunteers (25 participants, 4 sub-themes). - Indigenous health (24 participants, 2 sub-themes). - Access to health service delivery (11 participants, split int 2 sub-themes). - Economic impact of new programmes (4 participants, 1 sub-theme). - Outcomes impact of research partnerships (3 participants, 1 sub-theme).   Further questions were formulated:   - What strategies can be implemented to maximise data collection of rural injuries? - What injuries warrant injury prevention programmes? - What is effective in regard to community care and supports? - How can lack of available services be evaluated?   For the Eley & Baker study (2007)(2), 17 themes were discussed across the workshops. Workforce, mental health, access, and perceptions & expectations were raised in eight workshops. Workforce was ranked highest in ranking prioritisation of themes, second highest by number of comments, and highest overall ranking of themes. Aged care was mentioned in seven workshops, ranked second in prioritisation of themes, and highest by number of comments, but only sixth overall in terms of themes. Chronic disease was raised in four workshops, ranked lowly by prioritisation by themes, number of comments, and overall ranking of themes.(2)  The three rounds of focus group meetings each had their own purpose: idea generation, priority setting and choosing the top three themes for future research. For each round the research tool changed, commencing with an open question – What are the top priority research ideas you have for the Sunshine Coast Health Service District Mental Health Service? Participants in the second round of focus groups were asked to identify their most important ideas from a list of 16 ideas generated after the first round. The final round involved a shorter list of 10 items, from which participants were to identify their top three topics, which we termed themes.(7)  Ten problems related to current models of rural mental health and well‐being were identified. They are as follows:   - Rural communities are different from cities and are not homogenous; - The rural mental health system is not working; - Top‐down service models are based on urban assumptions; - Services are not based on needs; - The current forms of public financing are misaligned; - Fragmentation and competition hinder sustainable, robust service provision; - Structural inequity in mental health service provision is amplified in rural areas; - The rural mental health workforce cannot be a miniature version of that found in large cities; - While telehealth and online services should augment mental health services for all clients whether rural or urban, people with mental health challenges often need to speak in person with a health professional, and on some occasions, very quickly; and - Data sets are incomplete, disjointed and limited: many different and incompatible data sets are gathered and there is little data‐sharing or linkage.(8)   Ten solutions are proposed for rural mental health and wellbeing that together would benefit from robust testing and evaluation. They are as follows:   - Whole‐of‐community, place‐based approaches are promising; - New service models tailored to context must be considered; - Co‐designed bottom‐up processes should be pursued in collaboration with state and federal partners; - Holistic and integrated care models need testing; - New better‐aligned funding models are needed; - Whole of community approaches are needed, not pilot studies; - New rural workforce models are needed; - Digital technology contributes now and can do more as part of new systems; and - Enhance data collection, monitoring, linkage, analysis and planning.(8)   Ten principles to guide and support mental health research with rural communities.   - Equitable rural research funding; - Equality of status for all research stakeholders; - Co- creation, co- design, co- production and co-publication; - Continuing partnership with community; - Connection to the community; - Flexibility to respond; - Confidentiality and privacy; - Data sovereignty; - Evidence- informed knowledge to action; and - Enduring positive legacy and capacity building.(20)   Rural and agricultural research priority areas  Priority area 1: improving rural health services and care provision.  1. evaluating brief psychological therapy provisions for farmers and delivered at home or locally;  2. improving the integration of rural health services to be more flexible, responsive and collaborative; and  3. supporting health-care professionals working in rural and agricultural areas to obtain specialised training to communicate and work successfully within these communities.  Priority area 2: understanding the impact of rural living and working in mental health.  Priority area 3: strategies to improve rural community connectedness.  1. How clearly the purpose/intention of the research was explained.  2. The location of visits, and if the participant needed to travel.  3. The expected time needed to take part in the study.(19) |
| **F** | **Prioritization of research topics/questions** | |
| 23 | Describe methods and criteria for prioritizing research topics or questions | NGT was used to collate and categorise priorities. A facilitator collected ideas from each of the group. NGT allowed each of the group equal say. Ideas then analysed thematically by facilitators. Then all group members ranked ideas by order of importance.(1) For the Baker study(1), in the brainstorming session, a number of themes and sub-themes were identified:   - Health professional support and development (52 participants, 5 sub-themes). - Mechanisms for identifying local and regional needs (51 participants, 3 sub-themes). - Mental health (48 participants, 6 sub-themes). - Health and interaction with the environment (44 participants, 7 sub-themes). - Management of conditions for which little is known (34 participants, 3 sub-themes). - Post-acute care (29 participants, 6 sub-themes). - Evidence-based practice (27 participants, 2 sub-themes). - Health workforce, including volunteers (25 participants, 4 sub-themes). - Indigenous health (24 participants, 2 sub-themes). - Access to health service delivery (11 participants, split int 2 sub-themes). - Economic impact of new programmes (4 participants, 1 sub-theme). - Outcomes impact of research partnerships (3 participants, 1 sub-theme).   The workshop began with silent generation onto cards of key health issues. Ideas from the participants were then presented in plenary until all issues had been exhausted. Issues were then pooled into themes and participants were given the opportunity to rank the key issues.(2)  The three rounds of focus group meetings each had their own purpose: idea generation, priority setting and choosing the top three themes for future research. For each round the research tool changed, commencing with an open question – What are the top priority research ideas you have for the Sunshine Coast Health Service District Mental Health Service? Participants in the second round of focus groups were asked to identify their most important ideas from a list of 16 ideas generated after the first round. The final round involved a shorter list of 10 items, from which participants were to identify their top three topics, which we termed themes.(7)  Over 2 days, mental health researchers, academics, service providers, managers and commissioners identified problems and solutions: making use of national and international experience and evidence about service models, evidence from small‐scale local pilots and novel data analyses. Further teleconferences and five iterations of the draft statement were considered before reaching a final consensus. There was a strong consensus around the problems and potential solutions that would benefit from being examined robustly.(8)  In addition to research and service delivery, these experiences encompass activities such as consultations, workshops, focus groups, conferences, community meetings and producing publications, toolkits and reports conducted in the course of their work. The digital conferencing and RSTP provided a mechanism for the authors to offer a formal account of their experience and insights related to rural mental health research. The authors included mental health consumers, carers, researchers, clinicians, service managers and policy analysts working in diverse social, cultural and environmental contexts13 across the globe. The collective insights and themes were collated and distilled into core themes using expert- based consensus through digital conferencing. The themes were then shaped and rewritten once consensus was agreed via asynchronous communication.  Ten principles to guide and support mental health research with rural communities.   - Equitable rural research funding; - Equality of status for all research stakeholders; - Co- creation, co- design, co- production and co-publication; - Continuing partnership with community; - Connection to the community; - Flexibility to respond; - Confidentiality and privacy; - Data sovereignty; - Evidence- informed knowledge to action; and - Enduring positive legacy and capacity building.(20)   A short bespoke online survey (n = 29) was sent to all members of the VCSE incorporating a mix of selection and open comment questions relating to mental health research opportunity awareness, types of health studies of interest, key areas of research need, ideas to engage rural and agricultural communities in research and personal factors which encourage or prevent participation in research studies. Two in-person community consultations (n ¼ 10) were conducted to further discuss research priorities for the local rural and farming community, identification of barriers to engagement and ideas to engage farmers and their families in research studies.(19) |
| 24 | State the method or threshold for excluding research topics/questions | NGT allowed all workshop participants to have an equal say within the workshop situation. It also ensured that ideas of members were shared and connected on by all the participants in a non-threatening environment. Once all group members had an equal say about the topic, the ideas were thematically analysed by the facilitators and then all participants ranked each of the themes by importance (in this case, the participants were given five dots to place next to the themes important to them.(1)  Data were analysed by content analysis; thematic coding, comparison through the process of indexing and re-analysis through text search. In addition the data were quantified within and among both workshop and thematic issue according to prioritisation by participants, frequency of theme appearing and number of comments generated. Combining all these data generated a ranking of themes by importance.(2)  The three rounds of focus group meetings each had their own purpose: idea generation, priority setting and choosing the top three themes for future research. For each round the research tool changed, commencing with an open question – What are the top priority research ideas you have for the Sunshine Coast Health Service District Mental Health Service? Participants in the second round of focus groups were asked to identify their most important ideas from a list of 16 ideas generated after the first round. The final round involved a shorter list of 10 items, from which participants were to identify their top three topics, which we termed themes.(7)  Over 2 days, mental health researchers, academics, service providers, managers and commissioners identified problems and solutions: making use of national and international experience and evidence about service models, evidence from small‐scale local pilots and novel data analyses. Further teleconferences and five iterations of the draft statement were considered before reaching a final consensus. There was a strong consensus around the problems and potential solutions that would benefit from being examined robustly.(8)  The digital conferencing and RSTP provided a mechanism for the authors to offer a formal account of their experience and insights related to rural mental health research. The authors included mental health consumers, carers, researchers, clinicians, service managers and policy analysts working in diverse social, cultural and environmental contexts13 across the globe. The collective insights and themes were collated and distilled into core themes using expert- based consensus through digital conferencing. The themes were then shaped and rewritten once consensus was agreed via asynchronous communication.(20)  A short bespoke online survey (n = 29) was sent to all members of the VCSE incorporating a mix of selection and open comment questions relating to mental health research opportunity awareness, types of health studies of interest, key areas of research need, ideas to engage rural and agricultural communities in research and personal factors which encourage or prevent participation in research studies. Two in-person community consultations (n ¼ 10) were conducted to further discuss research priorities for the local rural and farming community, identification of barriers to engagement and ideas to engage farmers and their families in research studies.(19) |
| **G** | **Output** | |
| 25 | State the approach to formulating the research priorities | NGT was used to collate and categorise priorities. A facilitator collected ideas from each of the group. NGT allowed each of the group equal say. Ideas then analysed thematically by facilitators. Then all group members ranked ideas by order of importance.(1) For the Baker study(1), in the brainstorming session, a number of themes and sub-themes were identified:   - Health professional support and development (52 participants, 5 sub-themes). - Mechanisms for identifying local and regional needs (51 participants, 3 sub-themes). - Mental health (48 participants, 6 sub-themes). - Health and interaction with the environment (44 participants, 7 sub-themes). - Management of conditions for which little is known (34 participants, 3 sub-themes). - Post-acute care (29 participants, 6 sub-themes). - Evidence-based practice (27 participants, 2 sub-themes). - Health workforce, including volunteers (25 participants, 4 sub-themes). - Indigenous health (24 participants, 2 sub-themes). - Access to health service delivery (11 participants, split int 2 sub-themes). - Economic impact of new programmes (4 participants, 1 sub-theme). - Outcomes impact of research partnerships (3 participants, 1 sub-theme).   Data were analysed by content analysis; thematic coding, comparison through the process of indexing and re-analysis through text search. In addition the data were quantified within and among both workshop and thematic issue according to prioritisation by participants, frequency of theme appearing and number of comments generated. Combining all these data generated a ranking of themes by importance.(2)  The three rounds of focus group meetings each had their own purpose: idea generation, priority setting and choosing the top three themes for future research. For each round the research tool changed, commencing with an open question – What are the top priority research ideas you have for the Sunshine Coast Health Service District Mental Health Service? Participants in the second round of focus groups were asked to identify their most important ideas from a list of 16 ideas generated after the first round. The final round involved a shorter list of 10 items, from which participants were to identify their top three topics, which we termed themes.(7)  Over 2 days, mental health researchers, academics, service providers, managers and commissioners identified problems and solutions: making use of national and international experience and evidence about service models, evidence from small‐scale local pilots and novel data analyses. Further teleconferences and five iterations of the draft statement were considered before reaching a final consensus. There was a strong consensus around the problems and potential solutions that would benefit from being examined robustly.(8)  This declaration statement draws the authors' extensive collective knowledge built over many years of working with rural communities across nine countries. In addition to research and service delivery, these experiences encompass activities such as consultations, workshops, focus groups, conferences, community meetings and producing publications, toolkits and reports conducted in the course of their work.(20)  A multi-methods approach was used for data collection for this case study: an online survey (n = 29) and qualitative community group discussions (n = 10). Findings are presented descriptively and analysed with content analysis to generate indicative research priorities and recommendations for future mental health research study design.(19) |
| **H** | **Evaluation and feedback** | |
| 26 | Describe how the process of prioritization was evaluated | The workshop evaluation and feedback(1) focused on the following questions:  What did you enjoy most?   - Networking activities - Research ideas - Collaborative opportunities - Knowledge acquisition   Benefits to the organisation   - Networking activities - Research ideas - Collaborative opportunities - Knowledge acquisition - Advocacy for issues   Useful follow-up following workshop   - Outcome information - Further workshops - Report of discussion & findings - List of participants   In conclusion, the following recommendations are made as an outcome of this study:   1. Continue to disseminate these research findings so that they are widely known and become a blueprint for the strategic planning and development of the service. 2. Develop a research project to focus on emotional wellbeing and service quality and accountability. By engaging decision-makers in all stakeholder groups in a process whereby they are facilitated to refocus service challenges, reframing them into projects rather than problems, a solution-focused culture may begin to flourish. Outcomes such as self-efficacy, emotional wellbeing and increased satisfaction with service may be achieved. 3. Develop a research project to focus on consumer and carer involvement in care. An established methodology called Shared Decision Making (Deegan, 2010; Duncan, Best, & Hagen, 2010) is available, however it has not been explored for its fit with the Australian system. 4. Future research in the partnership should use a participatory methodology and mixed methods so that priority topics are advanced, embedded and incorporated in future research activities conducted by any stakeholder group.(7)   Engagement with rural and farming community members is essential to understand research priorities and design factors that may influence participation in future research projects. The work here suggests that researchers need to carefully consider how and where a study is promoted, the involvement of farmers and rural community members as co-researchers and key delivery factors such as time and location, confidentiality and continuity of personnel. In addition, understanding and addressing the thematic priorities and needs of the community is important when considering the research area itself.(19) |
| 27 | Describe how priorities were fed back to stakeholders and/or to the public; and how feedback (if received) was addressed and integrated | 43 topics were put forward by participants during the NGT plenary session.(1) From these, 12 themes were evident.   - Health professional support and development (52 participants, 5 sub-themes). - Mechanisms for identifying local and regional needs (51 participants, 3 sub-themes). - Mental health (48 participants, 6 sub-themes). - Health and interaction with the environment (44 participants, 7 sub-themes). - Management of conditions for which little is known (34 participants, 3 sub-themes). - Post-acute care (29 participants, 6 sub-themes). - Evidence-based practice (27 participants, 2 sub-themes). - Health workforce, including volunteers (25 participants, 4 sub-themes). - Indigenous health (24 participants, 2 sub-themes). - Access to health service delivery (11 participants, split int 2 sub-themes). - Economic impact of new programmes (4 participants, 1 sub-theme). - Outcomes impact of research partnerships (3 participants, 1 sub-theme). |
| **I** | **Implementation** | |
| 28 | Outline the strategy or action plans for implementing priorities | At the end of the session, there was general agreement in the group that the priority for regional research involving spatial information centred upon agricultural injuries and incapacitating disease patterns in various local geographic areas. It was expected that tracking what was happening locally, more effective prevention strategies and management policies could be developed.(1)  The study compared local priorities with national and state agendas. The fourth national mental health plan emphasises the importance of service quality initiatives such as service access and accountability, consumer and carer engagement, and social inclusion and recovery (Commonwealth of Australia, 2009). This local study reinforces the need for progress in these inter-related areas. The study has also provided an opportunity to reflect on the research partnership between the MHS (SCHSD) and the University of the Sunshine Coast. The experience has solidified our determination to work together, to maintain the relationship, to regularly clarify directions, to reflect on how well goals are being worked towards and to conduct future collaborative ventures in both practice development and research.(7)  1. We agreed to publish this statement with the intention of furthering discussion of the best ways of addressing poor access to care and poor mental health outcomes in rural and remote communities.  2. We also agreed to work towards a formal partnership of service providers Universities and NGOs to seek the goals we all desire—better rural mental health and well‐being and better access to rural mental health services.  3. We agreed to collaborate to attract funding to this high priority space with the goal of pursuing rigorous research that operates at scale, is translatable and can be used to improve the mental health of rural residents.(8)  The 10 principles and standards in this declaration will help guide researchers towards research that is ethical, sustainable and beneficial. But more than this, combining an approach of humility, respect and true partnership with a commitment to developing local capability will in itself, serve to enhance the mental health and well- being of rural communities in both the short term and the long term. We call on policy makers, governments, funders and research leaders to establish mechanisms to ensure rural mental health research adheres to the principles outlined in this declaration.  Ten principles to guide and support mental health research with rural communities.   - Equitable rural research funding; - Equality of status for all research stakeholders; - Co- creation, co- design, co- production and co-publication; - Continuing partnership with community; - Connection to the community; - Flexibility to respond; - Confidentiality and privacy; - Data sovereignty; - Evidence- informed knowledge to action; and - Enduring positive legacy and capacity building.(20)   Engagement with rural and farming community members is essential to understand research priorities and design factors that may influence participation in future research projects. The work here suggests that researchers need to carefully consider how and where a study is promoted, the involvement of farmers and rural community members as co-researchers and key delivery factors such as time and location, confidentiality and continuity of personnel. In addition, understanding and addressing the thematic priorities and needs of the community is important when considering the research area itself.(19) |
| 29 | Describe plans, strategies, or suggestions to evaluate impact | At the end of the session, there was general agreement in the group that the priority for regional research involving spatial information centred upon agricultural injuries and incapacitating disease patterns in various local geographic areas. It was expected that tracking what was happening locally, more effective prevention strategies and management policies could be developed.(1)  In summary the research achieved all its stated objectives of community engagement through workshops, identification of new research areas and identification of new researchers. In addition highlighting the views of health providers has raised the profile of important health issues. Already preliminary results of the project have been discussed within the State Parliament. Overall the outputs of the project are expected to contribute to improved health in rural and remote areas of Southern Queensland, and strengthen the collaborative relationship between the university and the local community.(2)  The study compared local priorities with national and state agendas. The fourth national mental health plan emphasises the importance of service quality initiatives such as service access and accountability, consumer and carer engagement, and social inclusion and recovery (Commonwealth of Australia, 2009). This local study reinforces the need for progress in these inter-related areas. The study has also provided an opportunity to reflect on the research partnership between the MHS (SCHSD) and the University of the Sunshine Coast. The experience has solidified our determination to work together, to maintain the relationship, to regularly clarify directions, to reflect on how well goals are being worked towards and to conduct future collaborative ventures in both practice development and research.(7) In conclusion, the following recommendations are made as an outcome of this study:  1. Continue to disseminate these research findings so that they are widely known and become a blueprint for the strategic planning and development of the service.  2. Develop a research project to focus on emotional wellbeing and service quality and accountability. By engaging decision-makers in all stakeholder groups in a process whereby they are facilitated to refocus service challenges, reframing them into projects rather than problems, a solution-focused culture may begin to flourish. Outcomes such as self-efficacy, emotional wellbeing and increased satisfaction with service may be achieved.  3. Develop a research project to focus on consumer and carer involvement in care. An established methodology called Shared Decision Making (Deegan, 2010; Duncan, Best, & Hagen, 2010) is available, however it has not been explored for its fi t with the Australian system.  4. Future research in the partnership should use a participatory methodology and mixed methods so that priority topics are advanced, embedded and incorporated in future research activities conducted by any stakeholder group.(7)  1. We agreed to publish this statement with the intention of furthering discussion of the best ways of addressing poor access to care and poor mental health outcomes in rural and remote communities.  2. We also agreed to work towards a formal partnership of service providers Universities and NGOs to seek the goals we all desire—better rural mental health and well‐being and better access to rural mental health services.  3. We agreed to collaborate to attract funding to this high priority space with the goal of pursuing rigorous research that operates at scale, is translatable and can be used to improve the mental health of rural residents.(8)  The 10 principles and standards in this declaration will help guide researchers towards research that is ethical, sustainable and beneficial. But more than this, combining an approach of humility, respect and true partnership with a commitment to developing local capability will in itself, serve to enhance the mental health and well- being of rural communities in both the short term and the long term. We call on policy makers, governments, funders and research leaders to establish mechanisms to ensure rural mental health research adheres to the principles outlined in this declaration.  Ten principles to guide and support mental health research with rural communities.   - Equitable rural research funding; - Equality of status for all research stakeholders; - Co- creation, co- design, co- production and co-publication; - Continuing partnership with community; - Connection to the community; - Flexibility to respond; - Confidentiality and privacy; - Data sovereignty; - Evidence- informed knowledge to action; and - Enduring positive legacy and capacity building.(20)   Engagement with rural and farming community members is essential to understand research priorities and design factors that may influence participation in future research projects. The work here suggests that researchers need to carefully consider how and where a study is promoted, the involvement of farmers and rural community members as co-researchers and key delivery factors such as time and location, confidentiality and continuity of personnel. In addition, understanding and addressing the thematic priorities and needs of the community is important when considering the research area itself.(19) |
| **J** | **Funding and conflict of interest** | |
| 30 | State sources of funding | The GLiMHR project of which this rapid evidence synthesis will inform was funded by the National Institute for Health and Care Research (Ref: NIHR207514) as part of their Mental Health Research Development Award Scheme. |
| 31 | Declare any conflicts or competing interests | There are no competing interests. |

# References:

1. Baker P, Hegney D, Rogers-Clark C*, et al.* Planning research in rural and remote areas. Rural Remote Health. 2004;4(2):1-11. Available from: <https://www.rrh.org.au/journal/article/266>.

2. Eley R, Baker P. Rural and remote health research: Key issues for health providers in Southern Queensland. Aust J Rural Health. 2007;15(6):368-72. 10.1111/j.1440-1584.2007.00918.x. Available from: <https://onlinelibrary.wiley.com/doi/pdf/10.1111/j.1440-1584.2007.00918.x>.

3. Fraser C, Judd F, Jackson H*, et al.* Does one size really fit all? Why the mental health of rural Australians requires further research. Aust J Rural Health. 2002;10(6):288-95. 10.1046/j.1440-1584.2002.00463.x. Available from: <https://onlinelibrary.wiley.com/doi/full/10.1046/j.1440-1584.2002.00463.x?sid=nlm%3Apubmed>.

4. Hourihan F, Kelly B. National health policy: What does this mean for rural mental health research? Aust J Rural Health. 2006;14(2):49-50. 10.1111/j.1440-1584.2006.00762.x. Available from: <https://onlinelibrary.wiley.com/doi/10.1111/j.1440-1584.2006.00762.x>.

5. Judd F. Progressing the agenda for rural mental health research. Rural Remote Health. 2006;6(3) Available from: <https://pdfs.semanticscholar.org/0c89/6bffc93542b321a9162d5b705a81119ebbf8.pdf>.

6. Judd F, Murray G, Fraser C*, et al.* The mental health of rural Australians: Developing a framework for strategic research. Aust J Rural Health. 2002;10(6):296-301. Available from: <https://onlinelibrary.wiley.com/doi/pdf/10.1046/j.1440-1584.2002.00438.x>.

7. McAllister M, Munday J, Taikato M*, et al.* Determining mental health research priorities in a Queensland region: An inclusive and iterative approach with mental health service clinicians, consumers and carers. Adv Ment Health. 2012;10(3):268-76. 10.5172/jamh.2012.10.3.268. Available from: <https://www.tandfonline.com/doi/abs/10.5172/jamh.2012.10.3.268>.

8. Perkins D, Farmer J, Salvador-Carulla L*, et al.* The Orange Declaration on rural and remote mental health. Aust J Rural Health. 2019;27(5):374-9. 10.1111/ajr.12560. Available from: <https://onlinelibrary.wiley.com/doi/full/10.1111/ajr.12560>.

9. Abraham I, Buckwalter J, Neese J*, et al.* Mental health of rural elderly: A research agenda for nursing. Issues Ment Health Nurs. 1994;15(3):203-13. 10.3109/01612849409009384. Available from: <https://www.tandfonline.com/doi/abs/10.3109/01612849409009384>.

10. Carpenter-Song E, Snell-Rood C. The changing context of rural America: A call to examine the impact of social change on mental health and mental health care. Psychiatr Serv. 2017;68(5):503-6. 10.1176/appi.ps.201600024. Available from: <https://psychiatryonline.org/doi/10.1176/appi.ps.201600024?url_ver=Z39.88-2003&rfr_id=ori:rid:crossref.org&rfr_dat=cr_pub%20%200pubmed>.

11. Handley T, Inder K, Kelly B*, et al.* Urban–rural influences on suicidality: Gaps in the existing literature and recommendations for future research. Aust J Rural Health. 2011;19(6):279-83. 10.1111/j.1440-1584.2011.01235.x. Available from: <https://onlinelibrary.wiley.com/doi/10.1111/j.1440-1584.2011.01235.x>.

12. Hartley D, Britain C, Sulzbacher S. Behavioral health: Setting the rural health research agenda. J Rural Health. 2002;18(5):242-55. 10.1111/j.1748-0361.2002.tb00934.x. Available from: <https://onlinelibrary.wiley.com/doi/abs/10.1111/j.1748-0361.2002.tb00934.x?sid=nlm%3Apubmed>.

13. Hauenstein E. Building the rural mental health system: from de facto system to quality care. In: Fitzpatrick J, Merwin E, editors. Annu Rev Nurs Res. New York, USA: Springer; 2014. 5. p. 143-74.

14. Keller P, Murray D, Hargrove D. A rural mental health research agenda: Defining context and setting priorities. J Rural Health. 1999;15(3):316-25. 10.1111/j.1748-0361.1999.tb00753.x. Available from: <https://onlinelibrary.wiley.com/doi/abs/10.1111/j.1748-0361.1999.tb00753.x?sid=nlm%3Apubmed>.

15. Rost K, Fortney J, Smith J. Use, quality, and outcomes of care for mental health: The rural perspective. Med Care Res Rev. 2002;59(3):231-65. 10.1177/1077558702059003001. Available from: <https://journals.sagepub.com/doi/epdf/10.1177/1077558702059003001>.

16. Thorndyke L. Rural women’s health: A research agenda for the future. Women's Health Issues. 2005;15(5):200-3. 10.1016/j.whi.2005.07.004. Available from: <https://www.sciencedirect.com/science/article/pii/S1049386705000599?via%3Dihub>.

17. Wagenfeld M. Mental health and rural America: A decade review. J Rural Health. 1990;6(4):307-22. 10.1111/j.1748-0361.1990.tb00685.x. Available from: <https://onlinelibrary.wiley.com/doi/abs/10.1111/j.1748-0361.1990.tb00685.x?sid=nlm%3Apubmed>.

18. Boyd C, Parr H. Social geography and rural mental health research. Rural Remote Health. 2008;8(1) Available from: <https://www.rrh.org.au/journal/article/804/>.

19. Teague B, Crouch-Read L, Haley E. Informing mental health research priorities and design with rural and agricultural communities: a public involvement consultation case study. Ment Health Soc Incl. 2025 10.1108/MHSI-01-2025-0003. Available from: <https://www.emerald.com/insight/content/doi/10.1108/mhsi-01-2025-0003/full/html>.

20. Roberts R, Munoz S, Thorpe K*, et al.* International declaration on rural mental health research: 10 guiding principles and standards. Aust J Rural Health. 2024;32(4):611-6. 10.1111/ajr.13167. Available from: <https://onlinelibrary.wiley.com/doi/10.1111/ajr.13167>.
