# Supplementary material for: Identifying the Mental Health Research Priorities in Rural Settings, With Implications for Coastal Communities: A Rapid Evidence Synthesis
Source: Aust J Rural Health. 2026 Mar 20;34(2):e70171. doi: 10.1111/ajr.70171 (PMC13003580; doi:10.1111/ajr.70171)
Supplement: Supplementary file 4 — Data S4: Supporting information. [file AJR-34-0-s001.docx]

**Appendix 1** **Priorities, sub-priorities, for rural mental health research and the frequency with which they were cited in the included publications**

| **Priority** | **Sub-priority** | **Number of instances** |
| --- | --- | --- |
| **Category 1 - Interventions** | | |
| **Priority 1 Rural mental health research should consider the diverse nature of rural communities. Interventions should be targeted to the needs of specific groups, e.g., indigenous and first nations.** | Inclusion of indigenous populations and focus on their health, including mental health. | 6 (23, 24, 36-38, 42) |
|  | Study of specific at-risk or disadvantaged rural populations and the relationship of their status to mental disorders should be a research priority. | 2 (33, 39) |
|  | Include those with lived experience of mental illness. | 1 (27) |
| **Priority 1 Total** | | **9** |
| **Priority 2 Rural mental health research should focus on early intervention in rural communities.** | Early intervention and prevention in rural communities is important. | 3 (28, 34, 38) |
|  | Interventions must be evidence based, population specific, provided proximate to where consumers live, acceptable to consumers and providers, integrated within settings, and sustainable in resource-poor rural communities and health care organisations. | 1 (35) |
|  | Universal interventions, provided to whole populations, involve implementation of measures regarded as desirable for everyone (e.g. seat belts, encouragement of exercise). | 1 (38) |
|  | Selective interventions are targeted at those individuals who have an increased risk of developing a disorder (e.g. parenting skills for unmarried teenage mothers, suicide prevention). | 2 (28, 38) |
|  | Indicated interventions are targeted at those already showing minor signs and symptoms of disorder. | 1 (38) |
|  | Focus on clinical efficacy, cost-effectiveness, and generalisability of interventions in nursing intervention models. | 1 (29) |
|  | What interventions can be developed to achieve comparable rates of sustained engagement in care between remote rural residents with psychiatric disorders and their urban counterparts? | 1 (40) |
| **Priority 2 Total** | | **10** |
| **Category 1 Total** | | **19** |
| **Category 2 – Space and Place** | | |
| **Priority 3** **Rural mental health research should seek to understand the influence of place-based variables on mental health.** | Need to investigate relationship between people, place, and health. | 1 (32) |
|  | In this context, place means more than an individual’s geographical location (urban, rural, remote, etc.) but also encompasses a person’s social position or social status within a hierarchically stratified structure of opportunities. | 1 (32) |
|  | These include physical environment, availability of healthy/unhealthy environments, sociocultural factors of neighbourhood or locale, representation of neighbourhood, services provided, lay systems of beliefs and behaviours, and labour markets. | 1 (32) |
|  | Cultural factors that influence help-seeking. | 2 (28, 34) |
|  | Are the results of clinical trials applicable regardless of residential environment? | 1 (41) |
|  | Outcomes-based research must be performed to evaluate the efficacy of rural implementation of social programmes and interventions designed for use in more urban settings. | 1 (41) |
|  | It would be useful to have studies of both rural/urban and intra-rural patterns of seeking help for mental disorder. | 2 (32, 42) |
| **Priority 3 Total** | | **9** |
| **Priority 4**  **The delivery of rural mental health services needs to be sensitive to local and regional needs.** | The delivery of rural mental health services responds to local and regional needs. | 2 (23, 28) |
| **Priority 4 Total** | | **2** |
| **Priority 5** **Rural mental health research needs to recognise the existence of, and respond to, the spatial mismatch between demand for mental health services in rural areas.** | The notion that rural areas are idyllic needs to be challenged. | 1 (23) |
|  | Geographical isolation can underscore mental health problems. | 1 (23) |
|  | Resources tend to be concentrated in urban areas, which can be difficult to access for residents of rural areas. | 1 (36) |
|  | Access to mental health services in rural areas is a priority area for research. Within this context, transport is important. | 2 (24, 28) |
|  | May help to highlight this spatial mismatch and inform how providers respond to it. | 1 ((23) |
| **Priority 5 Total** | | **6** |
| **Category 2 Total** | | **17** |
| **Category 3 - Stakeholder Engagement** | | |
| **Priority 6** **Engaging appropriate key stakeholders in rural mental health research.** | Different studies vary in their definition of whom they are depending on the focus of their studies. | 2 (23, 27) |
| **Priority 6 Total** | | **2** |
| **Priority 7 There is a need to better understand, using conventional and innovative research methods, the experiences of the rural mental health care workforce.** | Voice of rural mental health workforce is important. | 4 (24, 26, 31, 36) |
|  | Evidence-based treatments implemented by individuals other than doctoral level mental health care providers, supported by lay community workers, and delivered electronically are more likely to be sustained in resource-poor rural health care agencies. | 1 (35) |
|  | Upskill rural mental health workforce. | 1 (34) |
|  | More research still needs to be done to determine if these treatments result in quality outcomes and are sustainable in rural health care systems. | 1 (35) |
| **Priority 7 Total** | | **7** |
| **Priority 8 Rural mental health research methods should embrace participatory and co-design approaches at all stages of the research process to break down cultural barriers between researchers and rural communities.** | Participant observation, such as multiple case study designs and ethnographic research are necessary in order to break down the cultural barriers between researchers and rural residents. | 1 (35) |
|  | Need to link research to the production of knowledge and resources that benefit the community. | 1 (27) |
|  | Ethnography can reveal how rural residents respond to challenges in mental health in rural areas. | 1 (32) |
|  | Can inform interventions. | 1 (33) |
|  | Strengthen links with the community and involve them in participatory research. | 1 (37) |
| **Priority 8 Total** | | **5** |
| **Category 3 Total** | | **14** |
| **Category 4 – Improving Understanding** | | |
| **Priority 9** **Rural mental health research should improve the understanding of risk and protective factors for mental health in rural areas.** | Mental disorder in rural areas. Examine personal risk factors for mental health. | 1 (29) |
|  | Protective factor for people suffering from poor mental health in rural areas, e.g. accommodation, which should vary by age and needs. | 2 (32, 33) |
| **Priority 9 Total** | | **3** |
| **Priority 10 Future rural mental health research should focus on four key areas: pathways to care; prevention and early intervention; outcome of illness; and aetiology.** | Pathways to care. | 1 (38) |
|  | Prevention and early intervention. | 1 (38) |
|  | Outcome of illness. | 1 (38) |
|  | Aetiology. | 1 (38) |
| **Priority 10 Total** | | **4** |
| **Priority 11 It has to be recognised that health and mental health education and health and mental health literacy can help to improve awareness of how to manage conditions, as well as how to access services in rural areas. Education is needed on the trajectory of care, prevalence of illness, and service provision.** | Trajectory of care. | 1 (38) |
|  | Prevalence of illness. | 3 (25, 32, 34) |
|  | Service provision. | 2 (24, 25) |
| **Priority 11 Total** | | **6** |
| **Category 4 Total** | | **13** |
| **Category 5 – Standardising Data and Terminology** | | |
| **Priority 12 Future rural mental health research efforts need to use consistent terminology. This will facilitate a more meaningful synthesis of future research. Using ‘rural’ (or ‘urban’) as a unit of analysis can generate misleading or irrelevant data and ultimately lead to less effective policy.** | To be meaningful, research that attempts to use rurality as an explanatory variable must define which specific context variables are being used (and measured) to account for any rural effects. | 1 (39) |
|  | Develop consistent terminology through increased communication between researchers and clinicians. | 1 (33) |
| **Priority 12 Total** | | **2** |
| **Priority 13 There needs to be baseline epidemiological data that identifies the prevalence of rural mental health problems (and their correlation with factors such as accessibility/remoteness, employment, age, etc.) within rural areas.** | Datasets need to be more holistic and connected with the emphasis on data linkage. | 1 (26) |
|  | Enhance data collection methods. | 1 (26) |
|  | Participants should have a say in how data they produce will be used. | 1 (27) |
| **Priority 13 Total** | | **3** |
| **Priority 14 Future rural mental health research has to operationalise and standardise quality indicators on how rural mental health services are performing.** | While the values embodied in the quality indicators should be evident in all systems of care, how they are operationalised may need to vary across rural settings. | 1 (35) |
| **Priority 14 Total** | | **1** |
| **Category 5 Total** | | **6** |
| **Category 6 – Outreach** | | |
| **Priority 15 There is a requirement for gateway providers (outreach, rural GP surgeries, advocacy organisations, etc.) to engage in outreach with those at risk of mental health problems in rural areas to signpost them to providers of support.** | How do gateway providers affect take-up of mental health services in rural areas? | 1 (35) |
|  | Do seriously mentally ill individuals residing in remote rural areas receive poorer quality of care? | 1 (40) |
|  | If so, what are the relative contributions of the provider (e.g., provision of less evidenced-based care) and the patient (e.g., failure to stay engaged in care) to the poor outcomes observed in seriously mentally ill individuals living in rural areas? | 1 (40) |
| **Priority 15 Total** | | **3** |
| **Category 6 Total** | | **3** |
| **Category 7 – Collaboration** | | |
| **Priority 16 As an academic discipline, rural mental health research should establish and strengthen collaborative trans-disciplinary and trans-institutional networks.** | Continued research is needed to define and understand the differences (and commonalities) among urban and rural women, and among various subpopulations of rural women. | 1 (41) |
| **Priority 16 Total** | | **1** |
| **Category 7 Total** | | **1** |
| **Overall Total** | | **73** |
